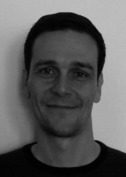# Bacterial Tropone Natural Products and Derivatives: Overview of their Biosynthesis, Bioactivities, Ecological Role and Biotechnological Potential

**DOI:** 10.1002/cbic.201900786

**Published:** 2020-05-08

**Authors:** Ying Duan, Melanie Petzold, Raspudin Saleem‐Batcha, Robin Teufel

**Affiliations:** ^1^ Faculty of Biology University of Freiburg 79104 Freiburg Germany

**Keywords:** natural products, roseobacticides, symbiosis, tropodithietic acid, tropolones

## Abstract

Tropone natural products are non‐benzene aromatic compounds of significant ecological and pharmaceutical interest. Herein, we highlight current knowledge on bacterial tropones and their derivatives such as tropolones, tropodithietic acid, and roseobacticides. Their unusual biosynthesis depends on a universal CoA‐bound precursor featuring a seven‐membered carbon ring as backbone, which is generated by a side reaction of the phenylacetic acid catabolic pathway. Enzymes encoded by separate gene clusters then further modify this key intermediate by oxidation, CoA‐release, or incorporation of sulfur among other reactions. Tropones play important roles in the terrestrial and marine environment where they act as antibiotics, algaecides, or quorum sensing signals, while their bacterial producers are often involved in symbiotic interactions with plants and marine invertebrates (e. g., algae, corals, sponges, or mollusks). Because of their potent bioactivities and of slowly developing bacterial resistance, tropones and their derivatives hold great promise for biomedical or biotechnological applications, for instance as antibiotics in (shell)fish aquaculture.

## Graphical abstract

**Accidents will happen**: The biosynthesis of bacterial tropone natural products depends on an unusual intertwining of primary and secondary metabolism. Remarkably, a shunt product arising from a “metabolic accident” of an aromatic catabolic pathway serves as universal precursor for tropone natural products.

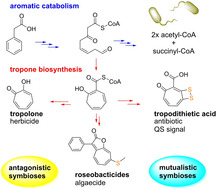

## 1 Introduction

Structurally diverse natural products are typically generated by dedicated secondary metabolic pathways that mostly operate in microbes and plants. Although not strictly required for growth, these specialized metabolites can improve the producing organism's chances of survival. For instance, natural products can serve as defensive or offensive weapons (antibiotics, toxins, virulence factors), signaling molecules (e. g., for quorum sensing (QS)), pigments (melanin, carotenoids, etc.), or siderophores to sequester metals.^1^ In addition, many symbiotic relationships involve secondary metabolites that can benefit (mutualistic symbioses) or afflict the host (antagonistic symbiosis). The bioactivity of these compounds depends on specific interactions with proteins or other macromolecules, albeit the exact molecular target is often challenging to determine. Typically, the biosynthesis of natural products (e. g., polyketides, nonribosomal peptides, or terpenoids) depends on characteristic enzymes for each compound class, which, in bacteria and fungi, are commonly encoded in gene clusters. In many cases, these specific gene signatures conveniently enable the *in silico* detection of putative (novel) natural product gene clusters and to some extent even the structural prediction of the respective compounds.^2^ On the other hand, noncanonical gene clusters/pathways and unforeseen enzyme reactions may impair detection and structural predictions.^3^

**Figure 1:**
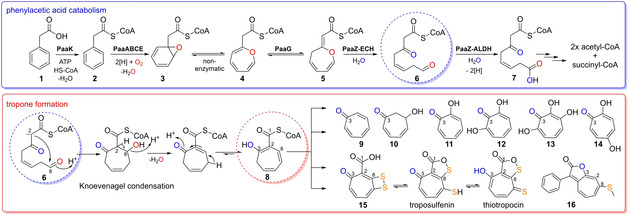
Overview of the bacterial catabolic pathway of **1** and formation of tropone natural products. Top: catabolic pathway converting **1** into **7**. Enzyme mechanisms and structures are shown in Figures 3–6. Final β‐oxidation‐like steps producing acetyl−CoA and succinyl−CoA are not shown in detail. A key intermediate for tropone formation is the highly reactive **6** (circled in blue), which arises from hydrolytic ring‐cleavage catalyzed by PaaZ−ECH (see text for details). Bottom: Spontaneous conversion of **6** into **8** (circled in red) that represents the proposed universal precursor for tropone natural products derived from **1** with some examples shown to the right. For tentative pathways of the different types of tropones, see Figures 7 and 8. Note that the carbon numbering of the compounds corresponds to **6** (not according to IUPAC rules).

A remarkable case of such a noncanonical biosynthetic route is found for bacterial tropone (**9**) natural products and derivatives thereof, including tropolone (**11**) and more complex sulfur‐containing metabolites such as tropodithietic acid (**15**). These non‐benzenoid aromatic compounds are of high ecological and pharmaceutical relevance and are mainly generated from phenylacetic acid (paa (**1**)) in bacteria, as outlined in this review (Figure 1).^4^ It is noteworthy that tropone‐moieties are also found in few actinobacterial aromatic polyketides (e. g., rubrolone),^5^, ^6^ which are synthesized differently and will not be further discussed here. In addition, tropones are produced by fungi and plants according to unrelated pathways. For an overview of plant and microbial tropone natural products including their chemical synthesis, we would like to refer the reader to other recent excellent reviews.^6^, ^7^, ^8^, ^9^ This review provides a comprehensive summary of the enzymology underlying bacterial **1**‐derived tropone biosynthesis, while also highlighting the compounds’ bioactivities, roles in symbioses, and possible biotechnological applications.

## 2 Biosynthesis and Occurrence of Bacterial Tropones and Derivatives

### 2.1 Sources of phenylacetic acid as precursor for the tropone backbone

Thiotropocine, which represents a tautomer of both **15** and troposulfenin (Figure 1), was isolated more than 35 years ago from *Pseudomonas* sp. CB‐104 and among the first reported bacterial tropone natural products.^10^ Meanwhile, numerous bacteria are verified producers, including Gram‐negative such as *Burkholderia* spp. or *Roseobacter* spp. but also Gram‐positive like *Streptomyces* spp..^6^, ^7^, ^11^ The biosynthesis of tropones is unusual and distinct from typical secondary metabolism, as the carbon backbone is generated by a primary catabolic pathway, i. e. the aerobic degradation of **1** (Figure 1).^12^

**Figure 2:**
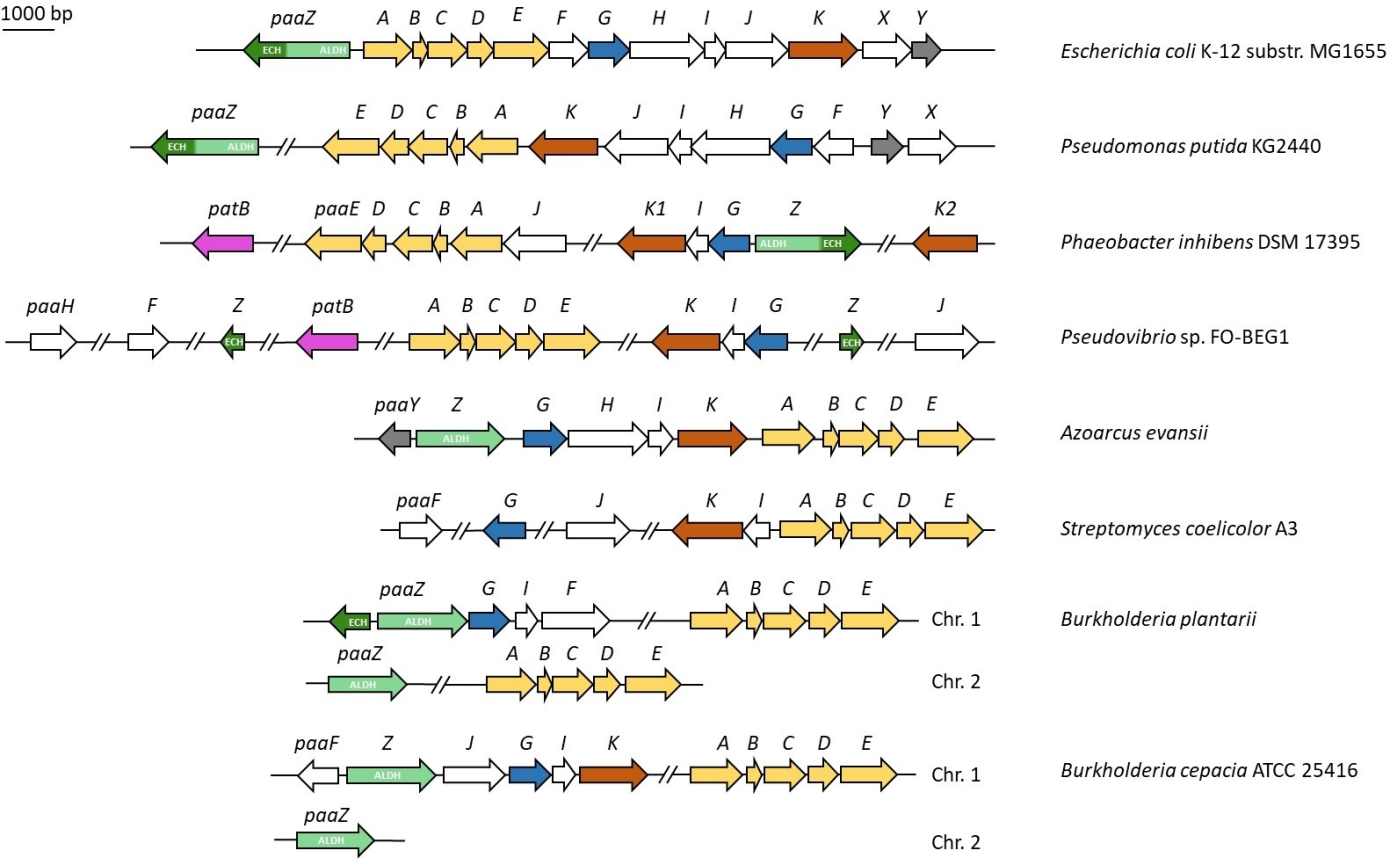
Selected bacterial **1** catabolic gene clusters. Important genes encoding enzymes required for tropone formation are color coded: *paaK* (brown), *paaABC(D)E* (yellow), *paaG* (blue), and *paaZ* (green; N‐terminal aldehyde dehydrogenase (ALDH) domain in light green, C‐terminal enoyl−CoA hydratase (ECH) domain in dark green). Note that many bacteria do not encode fusion protein PaaZ, but rather separate ECH and ALDH enzymes (ECHs are sometimes not present in the cluster but encoded elsewhere in the genome). PatB (pink) is most likely required for formation of the sulfur precursor of tropodithetic acid and roseobacticides (see text for details).

Compound **1** can either be obtained by direct uptake from the environment or by catabolism of diverse aromatic compounds (e. g., styrene, ethylbenzene or phenylalanine), which are first converted to **1** in peripheral (upper) pathways. This strategy reduces the amount of enzymes required for the subsequent concerted degradation of **1** in central (lower) pathways.^12^, ^13^ Alternatively, **1** is provided by the anabolic shikimate pathway from the precursors phosphoenolpyruvate and erythrose‐4‐phosphate that are converted to phenylpyruvate via various intermediates such as d‐arabino‐heptulosonic acid‐7‐phosphate (DAHP), shikimate, chorismate, and prephenate.^14^, ^15^ Bacteria that are specialized in tropone formation tend to synthesize the pathway precursors *de novo* according to the shikimate pathway and thus do not rely on exogenously provided aromatics. Typically, the genes required for **1** catabolism are clustered in bacteria (Figure 2), and the encoded enzymes that are required for the formation of the tropone backbone 2‐hydroxycyclohepta‐1,4,6‐triene‐1‐formyl−CoA (**8**) have been comprehensively studied. This includes the detailed structural and mechanistic investigation of the involved enzymes, as outlined in the following sections.

#### 2.1.1 Phenylacetyl−CoA synthetase PaaK

**Figure 3:**
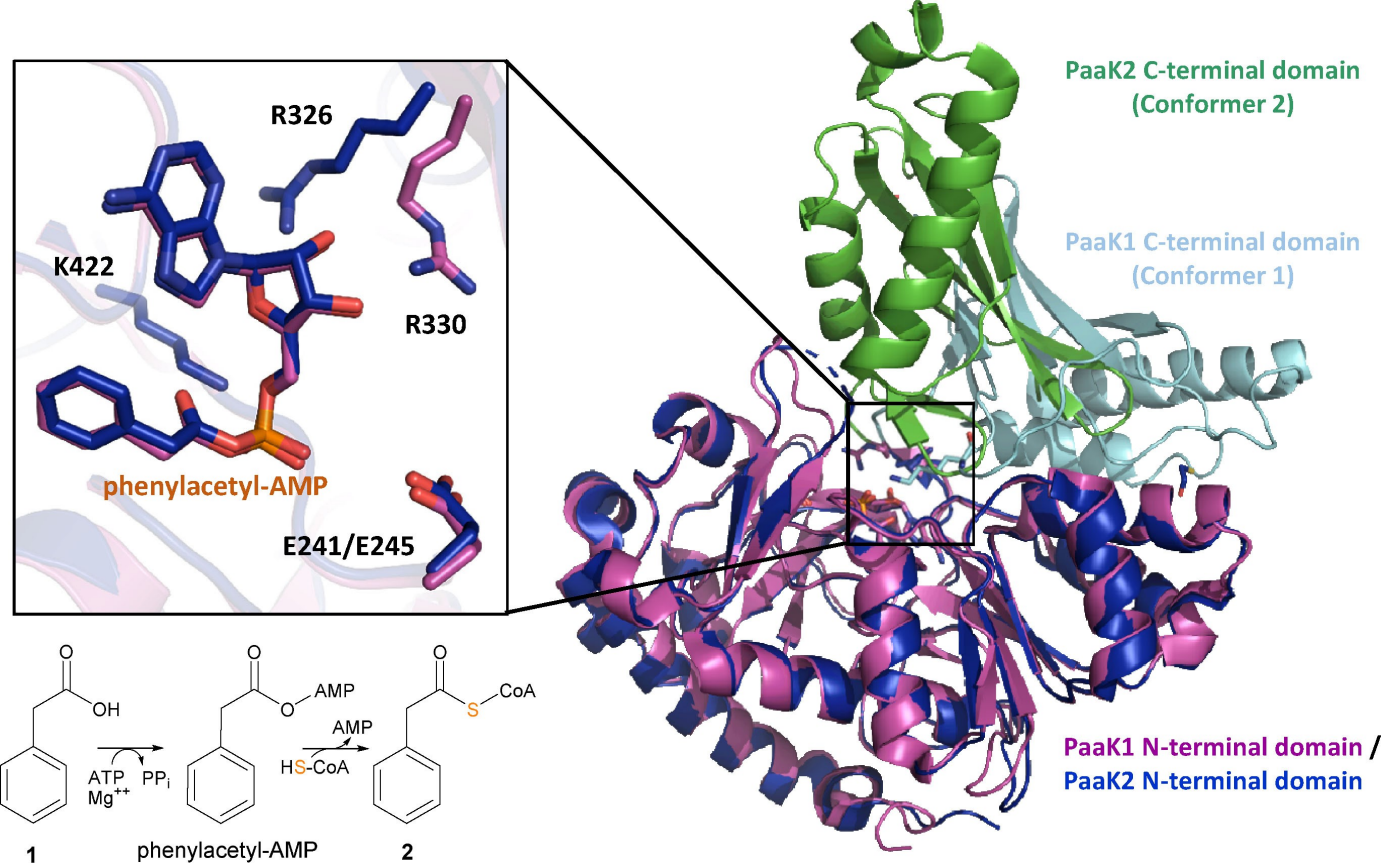
Crystal structures of PaaK paralogues from *Burkholderia cenocepacia*.^18^ Overlay of PaaK1 in conformation 1 adopted for adenylation (purple, mobile C‐terminal domain in light blue; PDB ID: 2Y4N) and PaaK2 in conformation 2 required for thioesterification (dark blue, mobile C‐terminal domain in green; PDB ID: 2Y4O). The inset shows a close‐up of the active site with critical residues and bound intermediate phenylacetyl−AMP (in yellow) as sticks. The reaction sequence for the conversion of **1** to **2** is shown at the bottom.

As a first step of the **1**‐catabolic pathway,^12^ **1** is converted to phenylacetyl−CoA (**2**). In **15**‐producing *Phaeobacter inhibens* (formerly *P. gallaeciensis* DSM 17395), it was also shown that phenylalanine can be directly converted to **2** via phenylpyruvate, thus skipping **1** as an intermediate during **15** biosynthesis.^16^ Typically, however, **1** is first ligated to coenzyme A (CoA) by phenylacetyl−CoA synthetase PaaK in an ATP and Mg^2+^‐dependent reaction with AMP and PP_i_ as co‐products.^17^ PaaK functional homologues are typically highly specific for phenylacetate and belong to a subgroup of the adenylate‐forming enzyme (AFE) family,^18^ whose members first catalyze the adenylation of a carboxylate group (yielding an acyl−AMP intermediate), before a conformational change of the C terminus enables thioester formation.^19^ Protein structures of PaaK have been elucidated from the pathogenic *Burkholderia cenocepacia* that causes infections in cystic fibrosis patients. Interestingly, *B. cenocepacia* encodes two paralogous enzymes (PaaK1 and PaaK2) for unknown reasons with slightly different aryl binding pockets resulting in distinct substrate scopes and kinetics (e. g., PaaK1 more readily accepted 3‐ and 4‐substituted substrates such as 4‐hydroxyphenylacetic acid). Several structures of protein‐substrate complexes were solved, for example, of PaaK1 in complex with ATP (conformation 1) or of PaaK2 with the bound phenylacetyl−AMP intermediate (conformation 2). Hence, similar to other members of the AFE family, a mobile C terminus undergoes conformational changes required for adenylation (conformation 1) and thioesterification (conformation 2; Figure 3). In addition, PaaK1 and PaaK2 feature a small N‐terminal microdomain, which may possibly promote the interaction with other catabolic enzymes.^18^

#### 2.1.2 Phenylacetyl−CoA epoxidase PaaABCE

**Figure 4:**
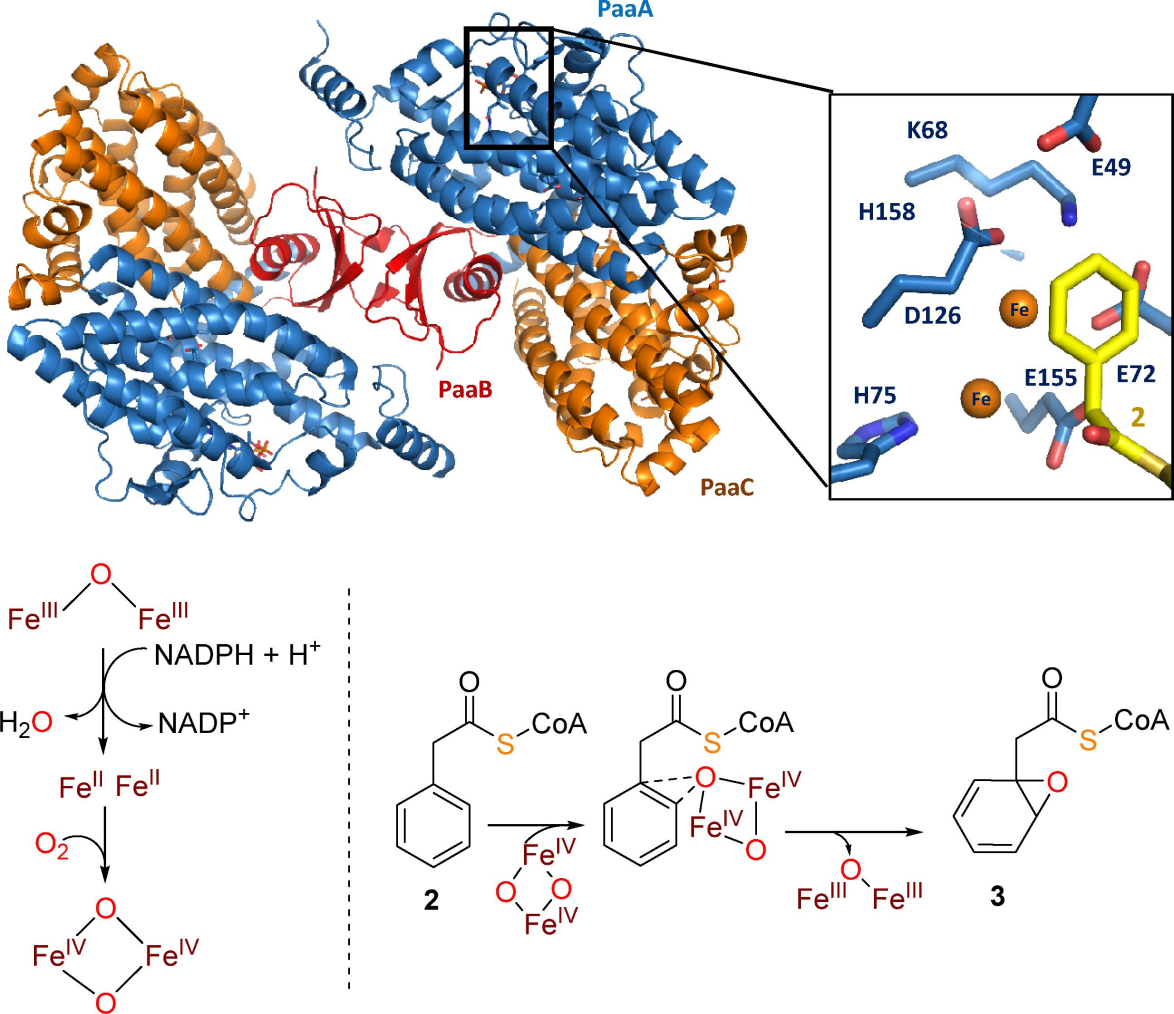
Composite representation of structures of the *E. coli* PaaAC subcomplex with bound **2** (PaaA in blue; PaaC in orange; PDB ID: 3PW1) and PaaB from *Klebsiella pneumonia* (red; PDB ID: 4IIT) that form heterohexamers (Paa(ABC)_2_). The inset shows the active site of PaaA with bound **2** (yellow sticks), critical amino acid residues (blue sticks) and two modeled iron ions.^22^, ^24^ Note that the interaction of the reductase subunit PaaE with the other subunits remains to be elucidated. Bottom: schematic representation of the proposed redox states of the diiron center and the reaction sequence catalyzed by PaaABCE.^21^

PaaABCE is an unusual diiron‐dependent multicomponent monooxygenase that epoxidizes the aromatic ring of **2**. PaaE is essential for reduction and thus activation of the diiron center from the catalytic subunit PaaA, whereas PaaC (a non‐iron containing homologue of PaaA) and PaaB presumably have structural, noncatalytic roles. In PaaA, **2** binds in a hairpin conformation with both ends (i. e., the adenine and phenyl moieties) pointing toward the active site, while the diphosphate group of the CoA moiety forms a bend close to the protein surface. As a result, the C1−C2 atoms of **2** are positioned next to the diiron center for epoxidation.^20^, ^21^ The two iron ions are likely complexed via the side chains of Glu42/Glu72/His75 and Asp126/Glu155/His158, respectively.^22^ Substrate oxygenation then likely proceeds analogous to the well‐studied soluble methane monooxygenase.^12^, ^21^ Accordingly, upon substrate binding, the diiron center ground state of PaaA is reduced to Fe^II^Fe^II^ by electrons delivered from PaaE. Through reaction of Fe^II^Fe^II^ with O_2_, the key oxygenating “diamond core” intermediate Fe^IV^(μ‐O)_2_Fe^IV[23]^ is formed that epoxidizes C1−C2 of **2** and thus affords the non‐aromatic 1,2‐epoxyphenylacetyl−CoA (**3**) (Figure 4).

High‐resolution structures are only available for the PaaAC subcomplex.^22^ However, from a low‐resolution crystal structure, electron microscopy and small angle X‐ray scattering, the role of PaaB could also be deduced. PaaA, B, and C accordingly assemble into a 2 : 2 : 2 heterohexamer ((PaaACB)_2_) with the PaaB dimer acting as bridge between the two PaaAC heterodimers (Figure 4).^24^ In contrast, the reductase component PaaE that belongs to the class IA reductases shows only weak interaction with other subunits and no structure of the whole complex has been solved yet.^12^, ^21^ PaaE features an NADPH‐binding domain and additionally contains a bound FAD cofactor as well as a ferredoxin‐like [2Fe‐2S] cluster. Based on structural modeling, PaaE may bind to PaaA in a way that orients the [2Fe‐2S] cluster toward the diiron site.^22^, ^24^ Electrons accordingly flow from NADPH via FAD and the [2Fe‐2S] cluster to PaaA.^21^ A conserved constellation of several amino acid residues of PaaA (Lys68, Glu49, Glu72 and Asp126 referred to as the “lysine bridge”) was furthermore proposed to participate in the relay of electrons from PaaE to PaaA's diiron center.^22^, ^24^ Originally, it was assumed that PaaD is also part of the monooxygenase complex. Yet, PaaD showed no interaction with other subunits and proved obsolete for catalytic activity *in vitro*. Instead, it was proposed that PaaD may be required for the maturation of the iron‐containing subunits A and/or E.^12^, ^22^

#### 2.1.3 1,2‐Epoxyphenylacetyl−CoA isomerase PaaG

**Figure 5:**
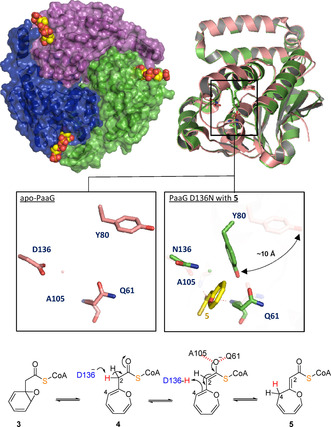
Crystal structures of PaaG from *T. thermophilus*. Top left: Surface representation of the trimeric PaaG−D136N variant in complex with product **5** (as spheres). Top right: Overlay of monomers of apo−PaaG (pink; PDB ID: 6SL9) with PaaG D136N‐**5** complex (green; PDB ID: 6SLA chain A). The insets show the active sites with critical residues and **5** as stick models. Upon ligand binding, the Tyr80 side chain undergoes a conformational change and expels water molecules by forming a lid over the active site. Bottom: Reaction sequence catalyzed by PaaG for the conversion of **3**/**4** into **5** including the catalytic aspartate side chain and thioester enolate transition states stabilized by hydrogen bonding interactions with Ala105 and Gln61. The shuttled proton is highlighted in red.

The PaaABCE‐produced intermediate **3** is highly unstable and prone to undergo epoxide ring opening and rearomatization by deprotonation from ring C2. The resulting 2‐hydroxyphenylacetyl−CoA then likely eliminates CoA by intramolecular acid anhydride formation.^21^ Moreover, as an intrinsic feature of epoxybenzene moieties (as part of **3**), a spontaneous and reversible electrocyclic rearrangement takes place to the respective oxepinyl tautomer (**4**).^25^ Possibly, **4** is then processed by PaaG,^12^, ^26^ which has recently been characterized in detail.^27^ PaaG is homologous to Δ^3^,Δ^2^‐enoyl−CoA isomerases and employs a catalytic aspartate side chain (Asp136 in *Thermus thermophilus*) for the reversible proton shuttling between C2 and C4 of **3**/**4**, which affords (*Z*)‐2‐(oxepin‐2(3*H*)‐ylidene)‐acetyl−CoA (“oxepin−CoA”, **5**). The thioester enolate transition state is thereby stabilized by H‐bonding with the main chain amides of conserved Gln and Ala residues in the “oxyanion hole” (Figure 5).^27^

Typically, Δ^3^,Δ^2^‐enoyl−CoA isomerases are auxiliary enzymes in fatty acid degradation required for the β‐oxidation of linear, unsaturated fatty acids with a double bond at an odd position. In fact, it was recently shown that PaaG also catalyzes such a reaction in a downstream step in **1** catabolism and converts *cis*‐3,4‐didehydroadipoyl−CoA into *trans*‐2,3‐didehydroadipoyl−CoA.^27^ PaaG complex structures with native ligands, however, revealed mobile elements that also allow the accommodation of the bulkier **3**/**4**. Moreover, a mobile tyrosine side chain (Tyr80 in *T. thermophilus*) expels water by forming a lid over the active site upon ligand binding and may thereby prevent uncontrolled and undesired water addition to the β‐carbon, while promoting isomerase functionality (Figure 5).^27^ PaaG thus elegantly acts as mediator between aromatic degradation and fatty acid metabolism by generating **5** that mimics a typical substrate for β oxidation (i. e., an α,β‐unsaturated CoA ester), which is prone to undergo subsequent ring hydrolysis at the β‐carbon.

#### 2.1.4 Bifunctional oxepin−CoA hydrolase and aldehyde dehydrogenase PaaZ

The last enzyme required for formation of the proposed tropone precursor is the ring‐cleaving PaaZ, which in most **1**‐degrading bacteria is a fusion protein consisting of a C‐terminal enoyl−CoA hydratase (ECH) domain and an N‐terminal NADP^+^‐dependent aldehyde dehydrogenase (ALDH) domain. In some bacteria, the *paa* cluster only codes for an NAD^+^‐dependent aldehyde dehydrogenase (e. g., PacL of *Azoarcus evansii*), whereas an ECH is encoded elsewhere.^14^ The hot dog‐fold PaaZ−ECH domain was originally named monoamine oxidase C (MaoC) in *Klebsiella aerogenes*, as the *paaZ* gene was found adjacent to *maoA*, *padA*, and *maoB*.^28^ MaoA functions as an amine oxidase that converts, for example, phenylethylamine to phenylacetaldehyde, which is then processed to **1** by phenylacetaldehyde dehydrogenase PadA. Hence, a peripheric (upper) pathway and the central (lower) **1** catabolic pathway are clustered in *K. aerogenes*, which resulted in the (misleading) annotation of the PaaZ−ECH domain and homologous proteins as MaoC‐like proteins that, however, rather act as *R*‐specific enoyl−CoA hydratases.^14^ These enzymes function analogously to the non‐homologous *S*‐specific enoyl−CoA hydratases.^29^ However, because the active centers of both types of hydratases are organized in a mirrored fashion, the resulting products have inverted stereochemistry.^30^

**Figure 6:**
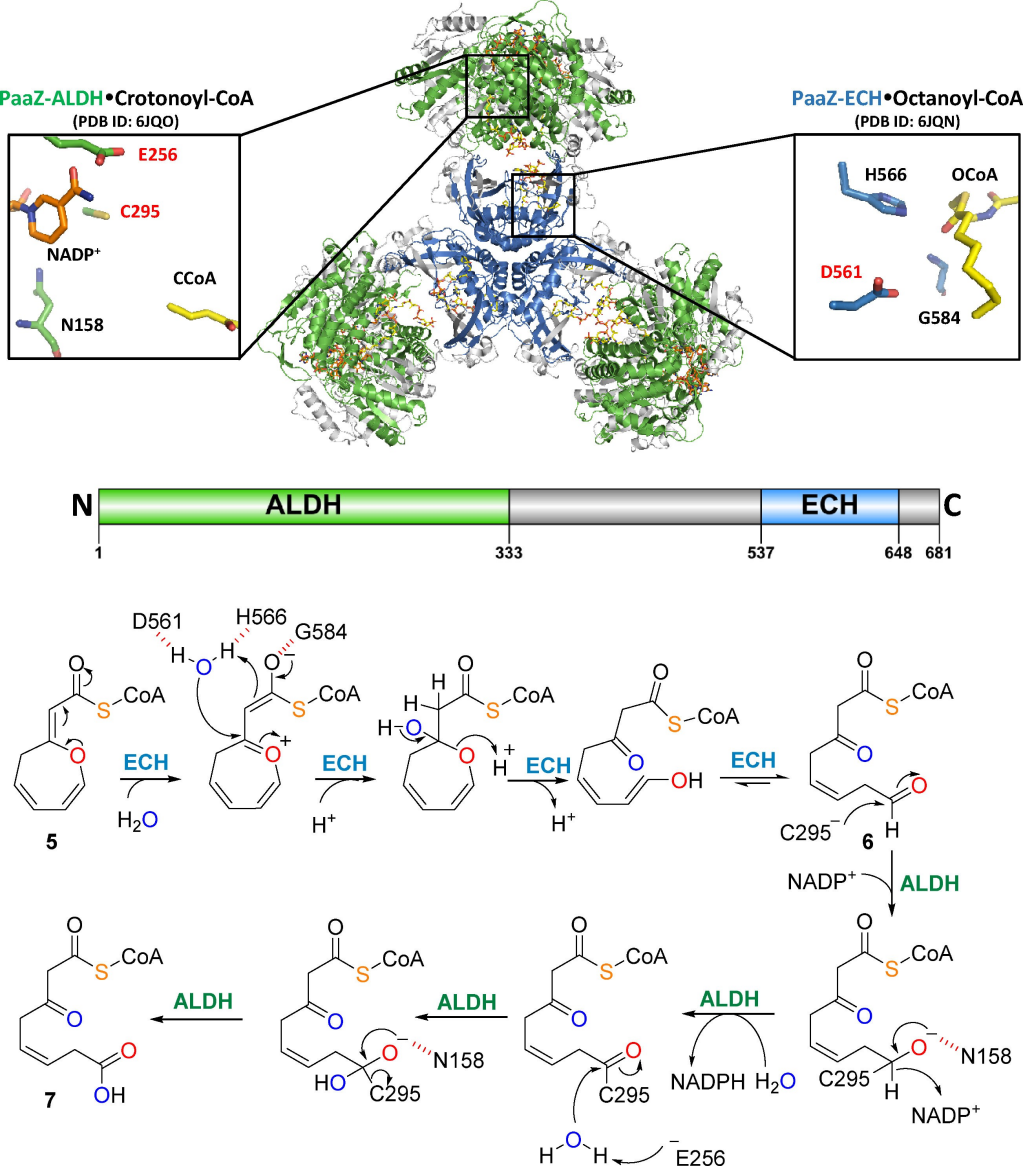
CryoEM structures of bifunctional PaaZ from *E. coli* with distinct ECH and ALDH domains.^31^ Overlay cartoon representation of hexameric, trilobed PaaZ (each lobe consists of an intertwined dimer). The substrate analogue crotonyl CoA bound in the active site of the ALDH domain was experimentally observed (green chains, PDB ID: 6JQN), whereas octanoyl CoA was modeled into the ECH active site (blue chains, PDB ID: 6JQO). Remaining protein segments are in gray. The domain organization of a PaaZ monomer is illustrated below with the same color code (according to Uniprot ID: P77455). The active site of the PaaZ−ECH domain of one monomer is close to the ALDH substrate‐binding tunnel of its intertwined partner monomer. The dehydrogenase domain active site comprises the catalytic nucleophile Cys295 and base Glu256. The insets display the active sites with critical residues and the substrate mimics as sticks (yellow). The proposed PaaZ catalytic mechanism is shown at the bottom (see text for details).

PaaZ from *Escherichia coli* K12 forms homohexamers with a trilobed architecture, in which each lobe consists of an intertwined PaaZ dimer with swapped ECH domains (all six ECH domains together form the inner core of the protein complex).^31^ PaaZ−ECH was shown to efficiently cleave oxepin−CoA, most likely via an acid‐base mechanism (Figure 6).^14^, ^30^ Ring cleavage by PaaZ−ECH is accordingly initiated by attack of a hydroxyl ion (i. e., generated with the help of the catalytic Asp561 and His566 side chains; numbering for these and the following residues according to *E. coli* K12) on the β‐carbon of the CoA−thioester enolate. Analogous to PaaG, the thioester enolate is stabilized by H‐bonding to the conserved Gly584 main chain. Both PaaG and PaaZ thus require CoA ester substrates for catalysis to enable the formation of enolate transition states, which may explain the atypical usage of CoA−esters in an aerobic aromatic degradation pathway. The newly introduced hydroxyl group is then converted to a ketone under concomitant C−O bond cleavage that opens the ring and ultimately affords the highly reactive 3‐oxo‐5,6‐dehydrosuberoyl−CoA semialdehyde (**6**) after keto‐enol tautomerism (Figure 6). However, no direct evidence for formation of unstable **6** could be provided so far, as chemical trapping of the aldehyde functionality with semicarbazide or phenylhydrazine failed.^14^

Compound **6** is then further oxidized by the N‐terminal NADP^+^‐dependent ALDH domain of PaaZ into 3‐oxo‐5,6‐dehydrosuberoyl−CoA (**7**). This step likely proceeds via formation of a covalent thiohemiacetal between the substrate and the Cys295 side chain, while the formed oxyanion is stabilized by H‐bonding interaction with the Asn158 side chain and the Cys295 main chain amide.^14^, ^32^ This enables subsequent hydride abstraction by NADP^+^, followed by addition of a nucleophilic hydroxide ion (generated by the Glu256 side chain that functions as catalytic base) to the transient thioester. Finally, the covalent bond is resolved and **7** is released. Further β‐oxidation‐like steps in the **1**‐catabolic pathway convert **7** into two molecules of acetyl−CoA and one succinyl−CoA, which are, however, not required for tropone natural product formation and will not be further discussed here.^12^, ^27^

#### 2.1.5 Formation of the tropone backbone via spontaneous Knoevenagel condensation

In previous studies, **8**, which represents the proposed precursor for bacterial tropone natural products (and most likely ω‐cycloheptyl fatty acids), was serendipitously discovered.^14^ In the course of the mechanistic investigation of PaaZ, an enzyme variant PaaZ−E256Q with abolished ALDH functionality surprisingly accumulated **8** rather than the expected **6**. Formation of **8** from **6** can be rationalized by a spontaneous Knoevenagel condensation, in which the nucleophilic C2 attacks the terminal C8‐aldehyde to form a seven‐membered ring. This is accompanied by the elimination of water and thereby generates **8** (Figure 1). This side reaction was in fact even observed in *in vitro* assays with the PaaZ wild type, leading to formation of minor amounts of **8** next to the main product **7**.^14^ These results suggested that **8** formation cannot be entirely prevented during **1** catabolism and bacteria seemingly adapted to this pitfall by developing different strategies. For instance, thioesterase PaaY was recruited for the pathway, which shows high specificity for **8** and thus prevents the potentially lethal depletion of the cellular CoA pool.^21^ PaaY thereby also ensures that the **1** catabolic pathway remains functional, as **8** (but not the CoA‐free 2‐hydroxycyclohepta‐1,4,6‐triene‐1‐formate) inhibits PaaZ.^21^

Moreover, the usage of a fusion protein for processing of **5** was proposed to be advantageous by allowing channeling of **6** from the ECH to the ALDH domains to expedite its oxidation and prevent excessive **8** formation.^14^ Recently, this hypothesis was corroborated by structural studies of PaaZ including small‐angle X‐ray scattering and cryogenic electron microscopy.^31^ Accordingly, the active site of a PaaZ−ECH domain was found closest to the ALDH substrate‐binding tunnel of its intertwined partner monomer rather than its own ALDH domain (Figure 6). Presumably, substrate channeling is then achieved via a set of conserved positively charged residues that promote the electrostatic pivoting of the CoA moiety and thus the transfer of **6** between the ECH and ALDH active sites of the partner monomers.^31^ However, many bacteria apparently found a more remarkable strategy for preventing CoA depletion and make use of **8** as precursor for tropone natural products (such as **15**) and ω‐cycloheptyl fatty acids.

### 2.2 Modification of the tropone backbone to natural products

Numerous bacterial tropone natural products are derived from **8**, which in turn is produced during (partial) **1** degradation. Some tropones are only slightly modified and may be formed from **8** without the requirement of additional dedicated enzymes, whereas more heavily modified tropolones (e. g., 7‐hydroxytropolone (**12**) or 3,7‐dihydroxytropolone (**13**)) and complex sulfur‐containing tropones (**15** or roseobacticide B (**16**) and congeners) require an extended set of enzymes, as outlined in the following sections. It is noteworthy that approximately 16 % of bacteria feature conserved genes of the **1** catabolic pathway and are thus among the potential producers of tropones. For example, a simple (point) mutation in PaaZ−ADLH could conceivably turn a **1**‐degrader into a producer of tropone natural products (as recently demonstrated for *E. coli*).^33^

#### 2.2.1 Non‐sulfur containing tropone and tropolone natural products

**Figure 7:**
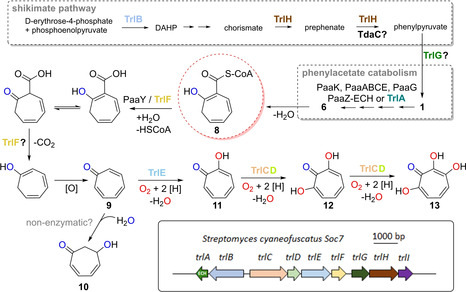
Tentative biosynthetic schemes of non‐sulfur tropone natural products (for **1** catabolic gene clusters, see Figure 2). Most likely, all compounds are derived from **8** (circled in red). Compounds **9** and **10** may be formed spontaneously following CoA hydrolysis of **8** by PaaY (or TrlF). Compounds **11**, **12**, and **13** require oxygenases such as TrlCDE encoded by the *trl* gene cluster (see bottom box). This cluster also encodes enzymes that likely boost the *de novo* formation of **1** via the shikimate pathway, that is, TrlB, TrlH and TrlG (similarly, TdaC might boost the biosynthesis of **15**, see also Figure 8). The proposed Trl enzyme functions were largely deduced from gene knock‐out studies and remain to be verified.

Simple derivatives of **8** are cyclohept‐4‐enol, cyclohept‐4‐enone, **9**, tropone hydrate (**10**), and the C2‐oxidized **11** that were previously isolated from phylogenetically diverse bacteria. For example, such compounds were detected among bacterial volatiles^34^ and some were clearly shown to be derived from **1**.^35^ In addition, **11** represents a virulence factor of several closely related terrestrial pathogenic *Burkholderia* species that are causal agents of bacterial panicle blight (also referred to as bacterial grain rot) and pose a global threat to rice production (see Section 4.1).^36^, ^37^ Insights into the generation of **9** came from studies with an *Azoarcus evansii* mutant strain with deleted *pacL* (encoding an ALDH responsible for the oxidation of **6** into **7** analogous to PaaZ−ALDH) that could no longer grow on **1** and instead accumulated **9** when cultivated in the presence of **1** in contrast to the wild type.^38^ This suggested that **9** can be formed without dedicated enzymes and possibly spontaneously from the free acid of **8**.^14^ Accordingly, following CoA hydrolysis by PaaY, (spontaneous) decarboxylation is then enabled by the β‐keto group, before autooxidation generates **9**. Most likely, **9** can then either undergo spontaneous or enzymatic water addition to form **10**, or become oxidized by a hydroxylase to **11** (Figure 7).

Interestingly, *Streptomyces* sp. were also reported as producers of tropones such as **13** first isolated from *S. tropolofaciens*.^39^ More recently, *S. cyaneofuscatus* Soc7 and *S. luteogriseus* Slg41 were also shown to produce **13** at high titers in addition to other tropolones such as **12** or 5‐hydroxytropolone (**14**;^40^ Figures 6 and 7). These Gram‐positive bacteria generate **13** via a dedicated gene cluster (named *trl*) that promotes the shunting of the **1** catabolic pathway.^40^ In total, nine additional genes (*trlA*–*trlH* and *trlR*) organized in three operons were found in this gene cluster. Heterologous production and gene deletion experiments confirmed **9**, as well as **11**, and **12** as intermediates in **13** biosynthesis. It is noteworthy that **12** was also among the first isolated bacterial tropolones originally obtained from *S. neyagawaensis*,^41^ which may thus harbor a similar gene cluster. TrlR is a predicted TetR family transcriptional regulator. Two enzymes (TrlB and TrlH) most likely boost production of pathway precursors by synthesizing phenylpyruvate via the shikimate pathway, which can be further converted to **1**. Presumably, TrlB functions as DAHP synthase, whereas TrlH is a bifunctional enzyme that converts chorismate via prephenate into phenylpyruvate (Figure 7). Hence, both TrlB and TrlH likely are involved in the *de novo* formation of **1** that can be further processed to **8**. Precursor supply possibly also involves TrlG, which was hypothesized to catalyze the conversion of phenylpyruvate into **1**.^40^

Notably, the two crucial enzymes for redirecting the metabolic flow of the **1** catabolic pathway are TrlA and TrlF. TrlA is a homologue of PaaZ−ECH that cleaves **5** and thereby steers formation of **8**, as an ALDH is not encoded in the cluster. In contrast, TrlF is a homologue of the thioesterase PaaY that specifically hydrolyses the CoA thioester of **8** 21 and may conceivably also promote the subsequent decarboxylation. Tropolone formation in the final pathway steps then involves flavin monooxygenase TrlE and two‐component flavin monooxygenase TrlCD for **11** and **13** formation, respectively. Other **1** catabolic enzymes required for **8** formation (PaaK, PaaABCE, and PaaG) are encoded elsewhere in the *Streptomyces* sp. genome.^40^

#### 2.2.2 Complex sulfur‐containing tropone natural products

##### 2.2.2.1 Tropodithietic acid biosynthesis

**Figure 8:**
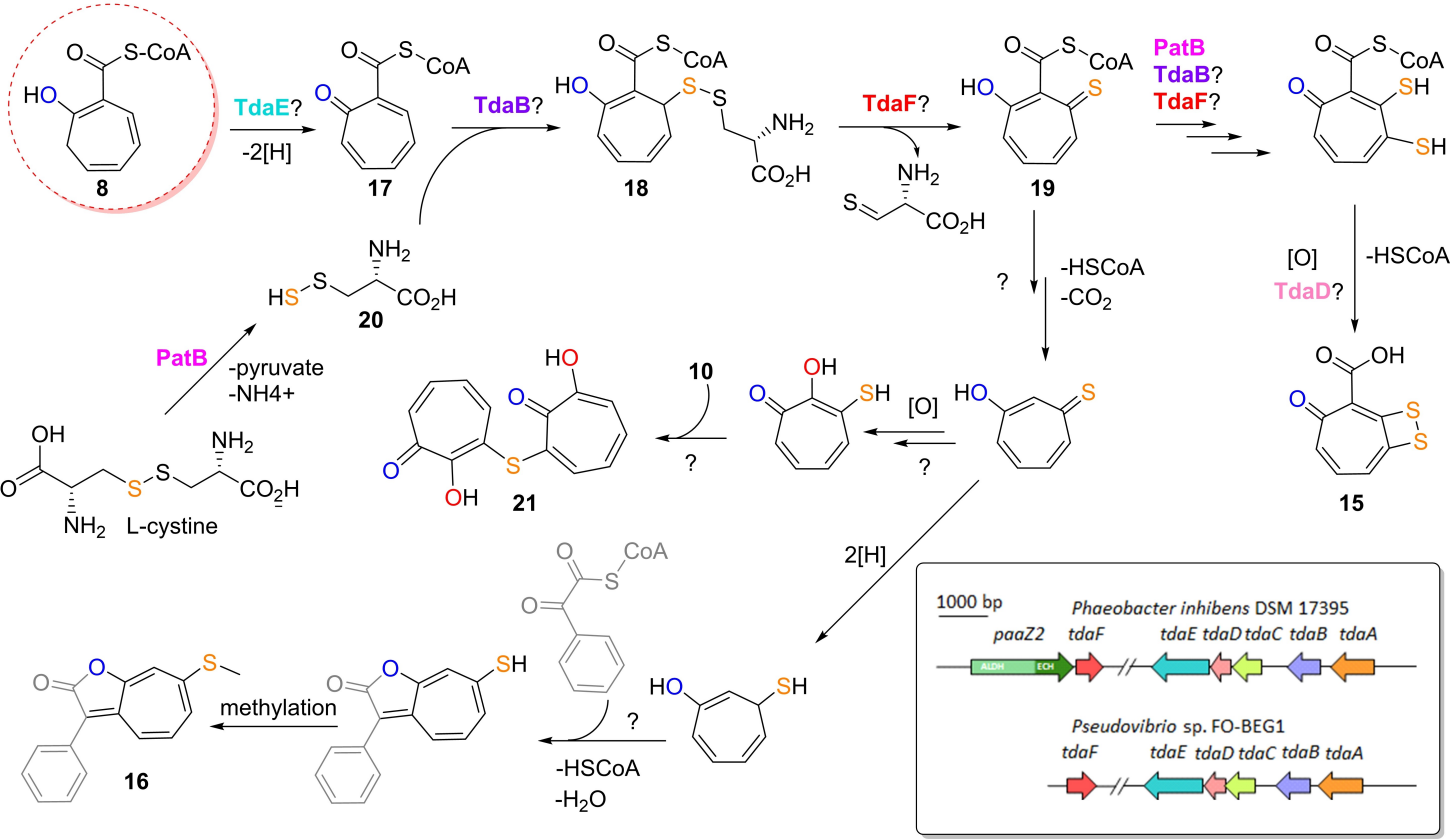
Tentative biosynthetic scheme and *tda* gene clusters for sulfur‐containing tropone natural products such as **15**, **16** and **21** (see Figure 2 for **1** catabolic gene clusters). Most likely, all compounds are derived from **8** (circled in red). All proposed enzyme functions remain (highly) speculative.

In addition to these simple derivatives, more complex, sulfur‐containing natural products such as **15** are produced from **8**. In contrast to the early **1** catabolic steps, however, the involved enzymes for downstream processing of **8** are only tentatively assigned. Early studies using random transposon insertion mutagenesis identified a total of 12 genes presumably required for **15** biosynthesis in *Ruegeria* and most likely in other genera of the *Rhodobacteraceae* (e. g., *Roseobacter, Phaeobacter, Pseudovibrio*).^4^ This included genes of the **1** catabolic gene cluster (*paaIJK,* which are differently annotated and equivalent to *paaCDE*, see Section 2.1.2.) as well as several genes (*tdaABCDEF)* of the newly discovered *tda* (tropodithietic acid) cluster that was found on a separate plasmid. Interestingly, aside from *paaZ1* located together with the **1**‐catabolic genes, *P. inhibens* featured a second copy (*paaZ2*) adjacent to the *tda* cluster (Figure 8). In contrast to PaaZ1−ALDH, the predicted PaaZ2−ALDH domain shows low similarity to other PaaZ homologues and carries a key mutation, i. e. the predicted catalytic cysteine residue required for formation of the thiohemiacetal is replaced with an arginine. PaaZ2 is thus likely only able to hydrolyze **5**, thereby promoting **8** formation.^42^ Hence, this represents an analogous strategy for pathway rerouting as employed by *Streptomyces* sp. (Section 2.2.1.).

The first step unique to **15** biosynthesis after **8** formation may be catalyzed by TdaE, which shows weak homology to acyl−CoA dehydrogenases that catalyze two‐electron oxidation reactions of CoA‐bound substrates. TdaE may accordingly oxidize **8** into troponylformyl−CoA (**17**; Figure 8).^43^ Notably, this reaction may also occur spontaneously, as implied by the observed formation of **9** in the *A. evansii pacL* mutant strain. The ring oxidation also increases the reactivity of the molecule, which becomes prone to undergo decarboxylation after CoA‐hydrolysis due to the presence of a β‐ketone.

An intriguing process in **15** biosynthesis is the sulfur incorporation and formation of the dithiet moiety. Most likely, the formation of the sulfur precursor depends on the cystathionine‐β‐lyase‐like enzyme PatB. First hints came from transposon mutagenesis of *P. inhibens* that indicated the requirement of *patB* for **15** biosynthesis.^44^ The important role of PatB was later corroborated by gene deletion of *patB* that abolished the production of **15** in *P. inhibens*, which could neither be salvaged by cysteine nor homocysteine addition.^42^ Feeding experiments in the same study with various [^34^S]‐labeled precursors, however, showed the incorporation of labeled sulfur into **15** from NaH^34^SO_4_, [^34^S]‐l‐cysteine, as well as its dimer [^34^S]‐l‐cystine.^42^ The following detailed *in vitro* studies of PatB compellingly demonstrated that l‐cystine is cleaved by PatB into pyruvate, ammonium, and the unstable *S*‐thio‐l‐cysteine (**20**), thus implying that this highly unstable compound might represent the sought‐for sulfur precursor. PatB displayed a broader substrate scope and could (among other sulfur‐ or selenium containing substrates) also less efficiently cleave l‐cystathionine into l‐homocysteine and ammonium, analogous to l‐cystathionine β‐lyases involved in the primary metabolism of sulfur.^45^

A plausible candidate for the incorporation of the PatB‐produced **20** is the predicted β‐etherase/glutathione‐S‐transferase TdaB. Typical β‐etherases catalyze the cleavage of β‐O‐4 aryl ether bonds between aromatic subunits in lignin^46^ via formation of a glutathione adduct, while glutathione‐S‐transferase are involved in the detoxification of xenobiotics and provide resistance against oxidative stress.^47^ These enzymes mediate a nucleophilic attack of glutathione on their substrates. Hence, instead of glutathione, TdaB may combine **20** with **17** to form **18** via a Michael addition that is probably enabled by the prior TdaE‐mediated oxidation step (Figure 8). Then, **18** could be oxidized into **19** by TdaF, which is a predicted flavin mononucleotide (FMN)‐dependent enzyme. TdaF resembles phosphopantothenoylcysteine decarboxylase from coenzyme A biosynthesis that catalyzes the FMN‐mediated oxidation of phosphopantothenoylcysteine, followed by decarboxylation and reduction of the generated intermediate with FMNH_2_, which thus regenerates FMN.^48^ In the case of TdaF, the resulting thioaldehyde co‐product may then be analogously decarboxylated and reduced to cysteamine by FMNH_2_, following the oxidation of **18**.^42^ Conceivably, **19** is once more processed by PatB, TdaB and TdaF to install the second sulfur, before a final spontaneous (or enzyme‐mediated) oxidation affords **15** (Figure 8).

As of yet, it remains unclear whether elimination of CoA during **15** biosynthesis occurs before or after formation of the dithiet moiety. Two enzymes are plausible candidates for catalyzing the required CoA−ester hydrolysis. First, PaaY, which is encoded by the **1** catabolic gene cluster. Because of the structural similarity to the native substrate **8**, it is highly likely that **17** and possibly later intermediates are also processed by PaaY. In addition, the *tda* cluster encodes another predicted thioesterase TdaD that could fulfill such a role, possibly in one of the final pathway steps.^42^

The role of TdaC remains obscure; it was proposed as dehydratase for formation of **8** from **6**.^43^ Yet, the dehydration occurs spontaneously as part of the Knoevenagel condensation without accumulation of any intermediates and an involvement of an enzyme thus appears redundant. As TdaC weakly resembles prephenate dehdratases of the shikimate pathway, it could instead be used for the conversion of prephenate into phenylpyruvate and thus provide precursors for tropone biosynthesis similar to TrlB and TrlH in *Streptomyces* sp. (Figure 7). The last encoded protein, TdaA, functions as a positive regulator of the *tda* cluster.^49^ In addition to **15**, an unusual antibacterial sulfur‐bridged tropolone dimer (**21**) was described from *Burkholderia cenocepacia* that likely also derives from **8** (Figure 8).^50^

##### 2.2.2.2 Roseobacticide biosynthesis

**Figure 9:**
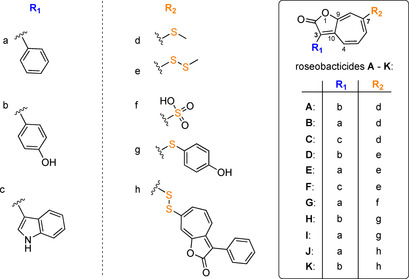
Structural variations of roseobacticides A–K.^51^

Interestingly, some **15** producers such as *Phaeobacter* sp. produce another class of tropone natural products with more complex carbon backbone referred to as the roseobacticides with at least 11 naturally occurring analogues (Figure 9).^51^, ^52^, ^53^ These compounds are key to a remarkable symbiosis of *P. inhibens* with the microalga *Emiliania huxleyi*. While **15** protects the algae from bacterial pathogens, a biosynthetic switch from formation of **15** to the algaecidal roseobacticides leads to host death (see Section 4.3.). Using transposon mutagenesis, it was found that roseobacticide biosynthesis mainly or exclusively depends on the same gene set as **15**, i. e. the **15** operon, the **1** catabolon, as well as *patB*.^54^ This is supported by another study that analyzed 33 genomes of members of the *Rhodobacteraceae*. Compound **15** and roseobacticides were produced by 27 and 18 species, respectively.^55^ Among these, all 20 strains of *P. inhibens*, *P. piscinae*, and *P. porticola* were producers of **15**. Strikingly, these **15**‐producing *Phaeobacter* sp. also generated roseobacticides with the exception of *P. piscinae* 27‐4^T^ strains and the only investigated strain of *P. porticola*. Other **15**‐producing bacteria (*Ruegeria* sp. and *Pseudovibrio* sp.) did not produce roseobacticides. Upon closer inspection of the genomes of roseobacticide producing *P. piscinae* M6‐4.2, *P. piscinae* 8‐1, *P. inhibens* DSM 17395 and *P. inhibens* DSM 26640 with the non‐producer *P. piscinae* 27‐4^T^, merely a few exclusive genes were found.^55^ Only two of these encode obvious putative enzyme candidates for roseobacticide biosynthesis that resemble a glutathione S‐transferase and a sulfurase. This may point toward a possible alternative sulfur incorporation mechanism in roseobacticide biosynthesis. Notably, the inability of *P. piscinae* 27‐4 to produce roseobacticides might also result from a transposable element that disrupted a gene encoding a putative oxidoreductase. This gene is part of a small cluster encoding in addition a transcriptional regulator, an endonuclease, as well as an aldehyde dehydrogenase.^55^ Further studies will be required to address these questions.

Taken together, roseobacticide biosynthesis most likely primarily depends on the genes from the *paa* and *tda* clusters required for **15** biosynthesis – possibly without requirement for additional enzymes. Hence, the *paa* and *tda* gene clusters may generate two types of molecules with substantially different structures and bioactivities.^54^ Yet, the bacterial producers somehow redirect the metabolic flow from formation of **15** to the roseobacticides during the symbiotic switch from mutualism to parasitism. This could be achieved, for example, by different regulation of **15**‐specific or roseobacticide‐specific genes, respectively. For instance, genes encoding enzymes required for roseobacticide side chain precursor formation could be induced or specific tailoring enzymes unique to roseobacticide biosynthesis that remain to be identified. Regulation may also take place on an enzymatic level, for example, via inhibition of enzymes catalyzing **15**‐specific biosynthetic steps.

The exact roseobacticide biosynthesis remains unclear, yet isotope labelling experiments shed light onto the precursors and possible pathway. Feeding experiments with [1,2‐^13^C_2_]**1** clearly established that the roseobacticide backbone^52^ (i. e., the seven‐membered carbon cycle) is derived from **1**, which is most likely converted to **8** before further modification steps that include CoA hydrolysis and decarboxylation (Figure 8). The backbone is then fused to a side chain (that derives from an aromatic amino acid) and thereby generates the central five‐membered lactone ring. The structure of the various roseobacticides implies that the side chains are derived from one of the three aromatic amino acids (Figure 9). Before being fused with the tropone‐moiety, the aromatic amino acids are further modified by decarboxylation and deamination, ultimately forming, for example, phenylglyoxylate from Phe that may be activated by CoA−thioester formation. Labeling experiments with *P. inhibens* DSM 17395 using deuterated and ^13^C‐containing aromatic amino acids verified this proposal and showed that intermediates of the Phe, Tyr or Trp catabolic pathways are employed for the generation of the aromatic side chains of the various roseobacticides A–K, whereas only Phe serves as precursor for the tropone moiety via intermediate **8** (Figures 8 and 9).^52^ Interestingly, the phenyl moiety of labeled phenylpyruvate – but not of labeled **1** – was verified as precursor for the aromatic side chain of **16**,^52^ which may be rationalized by an aminotransferase‐catalyzed conversion of Phe into phenylpyruvate that can be directly converted to **2** before further oxidation to phenylglyoxylyl−CoA, thus skipping **1** as intermediate. Phenylglyoxylyl−CoA could then undergo lactone‐ring forming fusion with the tropone moiety under water and CoA elimination. Finally, a methylation step could generate **16** (note that the exact structure of the tropone precursor is still unknown, yet labeling studies indicated that it cannot be tropone itself but rather a functionalized derivative thereof; Figure 8).^52^ Other roseobacticides harboring Trp‐ or Tyr‐derived side chains could be formed analogously via the respective α‐diketone CoA−thioester intermediates. Enzyme candidates for these steps are present in the genome of *P. inhibens* DSM 17395 but require experimental validation.^52^

Notably, the structural diversity in the roseobacticide family is imparted by various substituents at C3 and C7.^51^ As verified by the above described experiments, the C3‐diversity directly results from the different aromatic amino acid precursors, whose aromatic moieties are not further modified in the mature natural products.^52^ In contrast, C7 harbors sulfur adducts that display greater variety, including simple thiomethyl, methyl persulfide and sulfonate moieties. In addition, more substantial modifications can be found in form of *p*‐hydroxybenzenethiol or even a dimer of two roseobacticides connected via a disulfide bridge. As a challenge for future studies focusing of tropone natural product biosynthesis, it will be interesting to see at which stage the precursor for roseobacticides is branched off from **15** biosynthesis and how this process is controlled on a genetic and enzymatic level.

##### 2.2.2.3 Sulfur metabolism for tropodithietic acid and roseobacticide formation in members of the *Rhodobacteracea*

**Figure 10:**
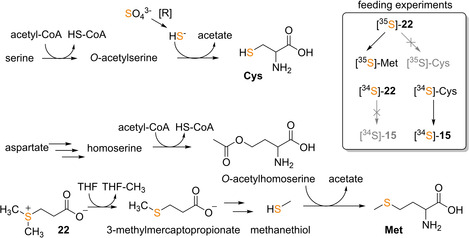
Proposed sulfur metabolism in members of the *Rhodobacteraceae*. Feeding experiments of *P. inhibens* cultures with isotopically labeled **22** and Cys suggested that Cys but not (**22**‐derived) Met serves as sulfur source for **15** biosynthesis. Cys can then be further converted to l‐cystine that serves as substrate for PatB, which may provide the sulfur precursor **20** for **15** biosynthesis (Figure 8).

Marine *Rhodobacteraceae* such as *Roseobacter* and *Phaeobacter* are well known for the production of numerous compounds with sulfur‐, disulfide‐, and thiol‐modifications.^4^, ^35^, ^56^ Hence, spontaneous addition of diverse thiols was originally proposed for roseobacticide biosynthesis,^51^ yet in the light of later results an involvement of PatB, possibly in conjunction with additional enzymes (see above), appears likely.^43^, ^54^, ^55^ Labeling experiments with [^34^S]‐Cys supplemented to *P. inhibens* DSM 17395 cultures clearly demonstrated the formation of [^34^S]‐labeled roseobacticides B and E that harbor thiomethyl and methyl disulfide moieties, respectively. Cys was speculated to be formed from the abundantly available food molecule dimethylsulfoniopropionate (DMSP, **22**),^52^, ^57^ which is produced by algae and corals in the natural environment of *Phaeobacter* sp. and readily metabolized.^56^, ^58^ Yet, isotope labeling experiments using radioactive [^35^S]‐**22** revealed the incorporation of [^35^S] mostly into Met, whereas labeled Cys was only detected in minor amounts.^59^ More importantly, labeling experiments using [^34^S]‐**22** and [^34^S]‐Cys fed to cultures of *P. inhibens* DSM 17395 and *P. inhibens* DSM 26640 clearly showed that only [^34^S] from Cys is incorporated into **15** (Figure 10). As **15** and roseobacticide formation could primarily rely on PatB for sulfur incorporation, Cys may exclusively serve as sulfur source for both types of natural products.

But how can this apparent disconnection between **22**/Met and **15**/Cys metabolism in *Rhodobacteraceae* be rationalized? In contrast to animals, the sulfur of Cys in plants and bacteria is typically not derived from Met but rather from sulfates that are intracellularly reduced to sulfides. Accordingly, cysteine biosynthesis involves the enzymatic conversion of serine to *O*‐acetylserine, which then reacts with sulfide under release of acetate to form Cys (Figure 10).^60^ In contrast to that, Met is derived from aspartic acid, while the sulfur may be provided either from Cys, methanethiol, or hydrogen sulfide. In *Rhodobacteraceae*, however, methanethiol is typically used, which it is a direct breakdown product of **22** 59 and reacts in a single step with *O*‐acetylhomoserine to Met.^61^ This suggests that the sulfur moieties of **15** and likely also of the roseobacticides are primarily derived from sulfates present in sea water (that are converted to Cys but not Met) rather than **22** (that is converted to Met but not Cys). It is noteworthy that this may not apply to all **15** and roseobacticide producing bacteria (Figure 10).

## 3 Modes of Action and Bioactivities of Bacterial Tropone Natural Products

Bacterial tropone natural products possess potent bioactivities. Clearly, decarboxylated simple tropones/tropolones (e. g., **9**, **11** or **13**) must differ in reactivity compared to sulfur‐containing **15** (and its tautomers) and roseobacticides, as a result of the respective distinct chemical structures and properties. The following sections highlight current insights into the underlying mode of actions (MoA) and bioactivities.

### 3.1 Tropodithietic acid

#### 3.1.1 Mechanism of action of tropodithietic acid

**Figure 11:**
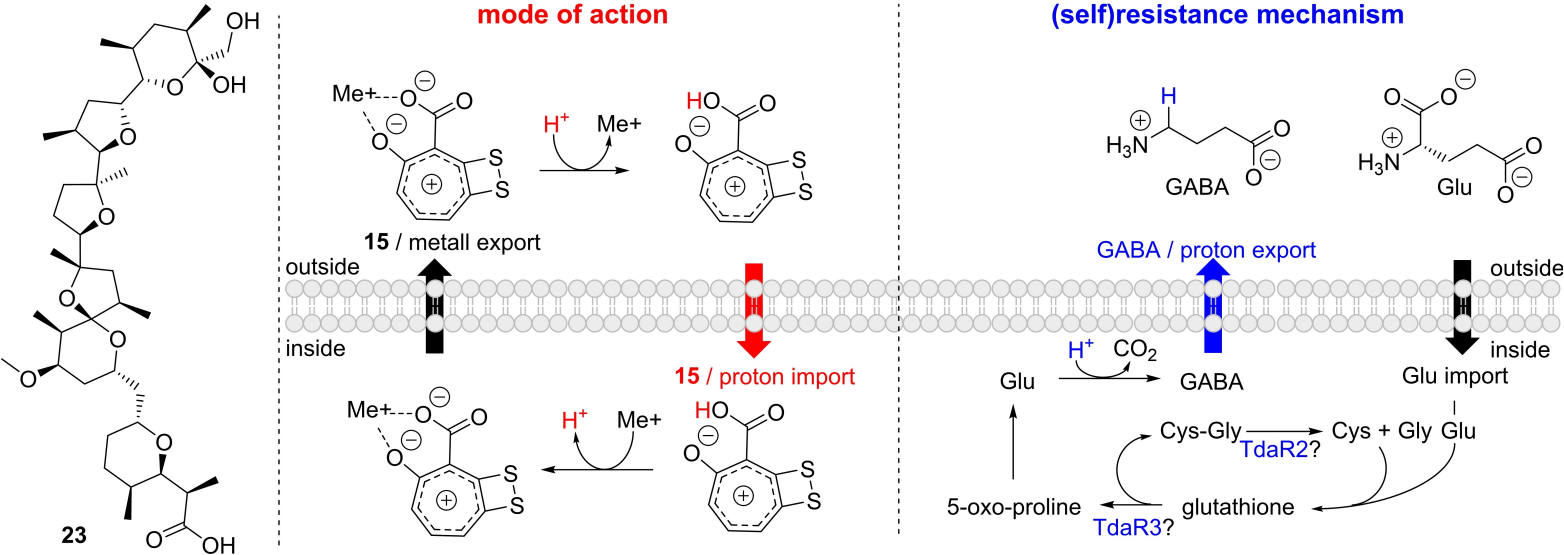
Proposed MoA of **15** and (self)resistance mechanism (figure modified after Wilson et al.).^63^ Compound **15** functions in a similar way to the more complex polyether natural products such as **23**. See text for details.

Compound **15** is the most thoroughly investigated bacterial tropone so far and recent studies also illuminated (part of) its MoA. Specifically, **15** was shown to act as potent membrane antiporter for protons in exchange for cytoplasmic cations (e. g., K^+^) ‐analogous to the structurally more complex polyether antibiotics (e. g., nigericin (**23**) or monensin, Figure 11). Hence, **15** collapses the cellular proton motive force (PMF) by affecting the transmembrane proton gradient (ΔpH) but not the membrane potential (Δ*Ψ*). These insights came from cytological profiling of **15**‐treated *E. coli* cells, which showed similar effects as when exposed to nigericin that exchanges protons for K^+^ ions, thus diminishing the ΔpH. Consistent with that, **15**‐treatment also affected PMF‐dependent cellular processes in *E. coli* such as flagella rotation. Compound **15** features a carboxyl side chain connected to a flat, seven‐membered ring with delocalized π‐bonds (comparable to the tropylium ion (C_7_H_7_^+^)).^62^ These structural features appear ideally suited for proton import across the membrane and cation export by complexation of metals.^63^ The dithietene moiety further promotes metal chelating properties by increasing the electron density of the tropylium oxide ring.^63^ Consistent with this proposed MoA, the bioactivity of a methylated **15** derivative (that can no longer translocate protons) was drastically reduced against the fish pathogen *V. anguillarum* compared to **15**.^64^

In addition to this MoA, recent studies showed that **15** efficiently complexes iron and presumably forms a [Fe^III^(**15**)_2_]_x_ complex.^65^ Although, **15** is not a true siderophore,^65^ it is quite possible that the iron‐chelating properties may further contribute to its bioactivity and/or provide a means to sequester iron from the environment.^65^ Consistent with that are recent studies that show an upregulation of iron‐uptake systems of *V. vulnificus* upon exposure to sublethal concentrations of **15**.^66^ Moreover, an oxidative stress response was triggered that induced genes required for formation of the cell envelope, cell wall and outer membrane (in line with studies showing toxicity and oxidative stress in neuronal cells upon exposure to **15**, see 5.1.2). Observations that **15** might be particularly effective against certain bacteria (e. g., *Vibrio* spp. among Gram‐negative strains, see below) possibly also point to a more complex MoA,^11^, ^66^, ^67^ as a **15**‐mediated collapse of the PMF should not be overly selective. However, the underlying mechanisms for this apparent selectivity require further investigation and remain controversial. For instance, while γ‐proteobacteria were first reported to be relatively tolerant toward **15**,^11^ later studies instead indicated a high sensitivity and no particular selectivity of **15** against different types of bacteria.^68^ The detailed MoA of **15** thus requires further investigation.^69^

#### 3.1.2 Minimum inhibitory concentration of tropodithietic acid against bacteria and anticancer activity

Consistent with the proposed MoA that collapses the PMF, **15** is not only bactericidal against Gram‐positive and negative bacteria,^67^ but also efficiently kills fungi, and even eukaryotic amoebae.^10^, ^63^ Minimum inhibitory concentration (MIC) values for both **15** and its tautomer thiotropocin are strongly pH‐dependent and have been reported for a broad range of Gram‐negative (e. g., *Salmonella enterica* ser. Typhimurium, *Enterobacter cloacea*, *Escherichia coli*, *Pseudomonas aeruginosa, Klebsiella pneumoniae*) and Gram‐positive bacteria (e. g., *Staphylococcus aureus* (MSSA and MRSA), *Enterococcus faecalis*, *Listeria monocytogenes*, *Bacillus subtilis*). Typically, MICs ranged between 14 and 118 μM for **15** 67, 70 and 7.4–118 μM for thiotropocin at pH 7. Notably, MIC values for thiotropocin were much lower at pH 9 and drastically higher at pH 5.^10^ Given the MoA of **15** that collapses the proton motive force,^63^ one can envisage that lower pH (acid stress conditions) further exacerbates the antibiotic effect and leads to accumulation of protons within the cells. In contrast, at elevated pH levels, **15** remains deprotonated in the extracellular environment and thus unable to transport protons into the cell. In general, Gram‐positive bacteria appeared to be more susceptible against **15** (note that the MICs for **15** from Table 1 of Porsby et al. were determined with impure extracts; the same report also provides accurate, much lower MICs for the purified compound).^67^ Interestingly, *Phaeobacter* sp. themselves are only protected from **15** when producing the compound, as the resistance mechanism is co‐regulated with **15** production (see below).

It is striking that among Gram‐negative bacteria, **15** seemed particularly efficacious against *Vibrio* sp. such as *V. vulnificus*, which are notorious (opportunistic) pathogens of marine animals. *V. vulnificus* even infects humans and may cause, for example, life threatening invasive sepsis or necrotizing wounds.^71^ For instance, **15** exhibited MICs of 1 and 3.9 μM^72^ against *V. vulnificus* DSM 10143 and *V. vulnificus* CMCP6 (or CMCP6_Rif1), respectively. In contrast, *V. parahaemolyticus* was more tolerant toward **15** with an MIC of 15.6 μM.^72^ Other studies reported MICs of 15.6 μM^69^ and 14 μM^67^ /19 μM^70^ for *V. vulnificus* CMCP6 and the fish pathogen *V. anguillarum* 90‐11‐287, respectively. Low MICs of 2.3 μM were also determined for the coral pathogens *V. coralliilyticus* and *V. owensii*.^73^ Other pathogenic bacteria *V. anguillarum* NB10 (infecting fish), *Roseovarius crassostreae* CV919, and *V. tubiashii* RE22 (both infecting mollusks) were affected by **15** with MICs of 5.9 μM, 23.6 μM, and 29.5 μM, respectively.^74^ Consistent with that, recent studies showed that fast‐growing opportunistic pathogens (*Vibrio* spp. and *Pseudoalteromonas* spp.) were drastically affected by the presence of *P. inhibens* in microbial communities associated with the marine eukaryotes *E. huxleyi* and *Ostrea edulis* (European flat oyster).^66^ Interestingly, structure‐activity relationship studies using chemically synthesized analogues of **15** including halogenated derivatives showed that **15** was generally the most potent antibiotic when tested against *S. aureus* and *V. anguillarum* 90‐11‐287.^70^

Given the MoA, it is not surprising that **15** also exhibits broad anticancer activities, for example, against renal cancer cells, central nervous system cancer cells, melanomas, colon cancer cells, and lung cancer cells. In particular, **15** exhibited LC_50_ values in the low μM range (5–7 μM) against central nervous system cancer and renal cancer cell lines; inhibitory effects were already observed at IC_50s_ of 1–3 μM. In contrast, noncancerous epithelial cells tolerated higher levels with an IC_50_ of roughly 19.5 μM.^63^

#### 3.1.3 Resistance against tropodithietic acid

Wilson *et al*. also described an operon of *P. inhibens* DSM 17395 that confers resistance against **15** and thus protects the native producers to a certain degree.^63^ A combination of bioinformatic and biochemical analyses revealed the involvement of the γ‐glutamyl cycle in this resistance mechanism. Specifically, three genes (*tdaR1*–*R3*) were identified adjacent to the *tda* operon and found to be co‐regulated with the **15** biosynthetic cluster by the transcription regulator TdaA.^49^ This is consistent with previous studies showing that *P. piscinae* 27‐4 is susceptible to its own antibiotic **15** when not in the late exponential phase during which **15** is produced.^67^ When TdaA was inactivated, the native producer was significantly more susceptible toward its own antibiotic **15**, most likely as *tdaR1*–*R3* were not upregulated.^63^ This hypothesis was corroborated by experiments with *E. coli* that contained an inducible plasmid harboring *tdaR1–tdaR3*. In contrast to control cells, *E. coli* with induced *tdaR1–R3* were more resistant against **15**, recovered faster, and ultimately reached higher cell densities in liquid culture.^63^

The role of TdaR1 that is probably a transmembrane protein (like TdaR2) could not be predicted based on bioinformatic analysis. In contrast, TdaR2 shows similarity to Zn^2+^‐dependent endopeptidases that cleave Gly−Gly targets, whereas TdaR3 is homologous to the γ‐glutamyl‐cyclotransferase ChaC with high specificity toward glutathione.^75^ The *cha* operon and ChaC of *E. coli* are involved in cation‐proton exchange, and TdaR3 could thus conceivably counteract the proposed MoA of **15** (Figure 11). Similar to ChaC, TdaR3 may thus convert glutathione into a Cys−Gly dipeptide and, crucially, 5‐oxo‐proline that can be further processed under uptake of a proton to γ‐aminobutyric acid (GABA). GABA is subsequently exchanged for an extracellular Glu, thus leading to proton export – a mechanism that is also part of the Glu‐dependent acid‐stress response in *E. coli*.^63^ In addition to providing resistance against **15**, the induced *tdaR3* also promoted the growth and recovery of *E. coli* cells exposed to sublethal concentrations of the protonophore carbonyl cyanide *m*‐chlorophenyl hydrazin (CCCP). Finally, TdaR2 could be involved in hydrolysis of the Cys−Gly dipeptide arising from TdaR3‐mediated glutathione cleavage (Figure 11). However, despite this resistance mechanism that requires further experimental validation, *P. inhibens* DSM 17395 was affected by **15**, resulting in reduced growth rate and biomass yield of the **15**‐producing wild type compared to non‐producer mutant strains. The growth defect is likely caused by increased energetic costs due to the partial collapse of the PMF.^76^ Taken together, the vast majority of bacterial strains appear highly susceptible toward **15** (with possible exceptions found in sponge microbiota, see 4.2.2), while **15**‐producers can achieve a moderate level of tolerance.

In contrast to **15**, the MoA of the roseobacticides has not been elucidated to date and warrants further investigation. These potent algaecides cause cell death of *E. huxleyi* in the nM range and affect at least two other microalgae.^51^

### 3.2 Non‐sulfur containing tropolones and derivatives

Among the non‐sulfur containing bacterial tropone natural products, tropolones are particularly relevant in terms of bioactivity. The majority of previous studies were conducted with plant or fungal tropolones, which exhibit antimicrobial, antiviral, anticancer, insecticidal, and anti‐inflammatory activities.^6^, ^9^, ^77^, ^78^ Similar bioactivities can be expected and have been reported to some extent for their bacterial counterparts.^39^ For instance, **13** from *S. tropolofaciens* showed potent cytotoxicity against cultured melanoma cells and prolonged the life span of mice with melanoma.^39^ Notably, additional oxygen atoms attached to the tropone backbone (in particular when three adjacent oxygen atoms are present as in **13** and other hydroxytropolones) proved critical for the metal‐chelating properties (Mg^2+^, Zn^2+^, Cu^2+^) that are likely responsible for the observed broad bioactivities and interference with metalloenzyme activity.^6^, ^79^, ^80^ Hydroxytropolones inhibit bimetallic enzymes competitively or uncompetitively, for example, inositol monophosphatase, alkaline phosphatase and dopamine β‐monooxygenase.^80^ Many of the targeted enzymes are of therapeutic interest, for example, Zn^2+^/Cu^2+^‐dependent metalloproteases such as the anthrax lethal factor, thermolysin, or matrix metalloproteases.^6^, ^9^, ^78^ Because of these promising bioactivities, tropolones have been investigated as therapeutic drug leads.^79^, ^80^, ^81^ For example, human immunodeficiency virus type I reverse transcriptase (HIV‐RT) is a key target to combat HIV infections and features two Mg^2+^ ions 3.7 ångströms apart in the active site. Compound **13** and derivatives that maintained the metal‐chelating properties were shown to drastically affect HIV‐RT activity, most likely by complexing both Mg^2+^ ions.^79^, ^80^

Interestingly, **12** produced by *S. neyagawaensis* was also shown to increase the efficacy of aminoglycoside antibiotics when administered to resistant bacterial strains. This effect was due to the competitive inhibition of aminoglycoside 4′‐O‐adenylyltransferase that normally confers resistance.^41^ Presumably, this effect was caused by the interference of **12** with two catalytically important Mg^2+^ ions in the active site that also impeded the binding of ATP.^82^ For potential further drug development and application of hydroxytropolones, however, toxicity issues would have to be addressed first.^79^ While three neighboring oxygen atoms are important for chelating of two suitably positioned metal ions by hydroxytropolones, tropolones such as **11** that feature only two neighboring oxygens cooperatively complex a ferric iron (Fe^3+^) and form a red precipitate.^83^ These iron‐binding properties are essential for the toxicity of **11** and its role as virulence factor (see Section 4.1.). It is noteworthy that in addition to metal complexation, tropolones are redox active and possibly interfere with respiratory chains.^6^

## 4 Ecological Role and Symbioses Involving Bacterial Tropones

### 4.1 Non‐sulfur‐containing tropolones as virulence factors and quorum sensing signals from pathogenic *Burkholderia* sp.

**Figure 12:**
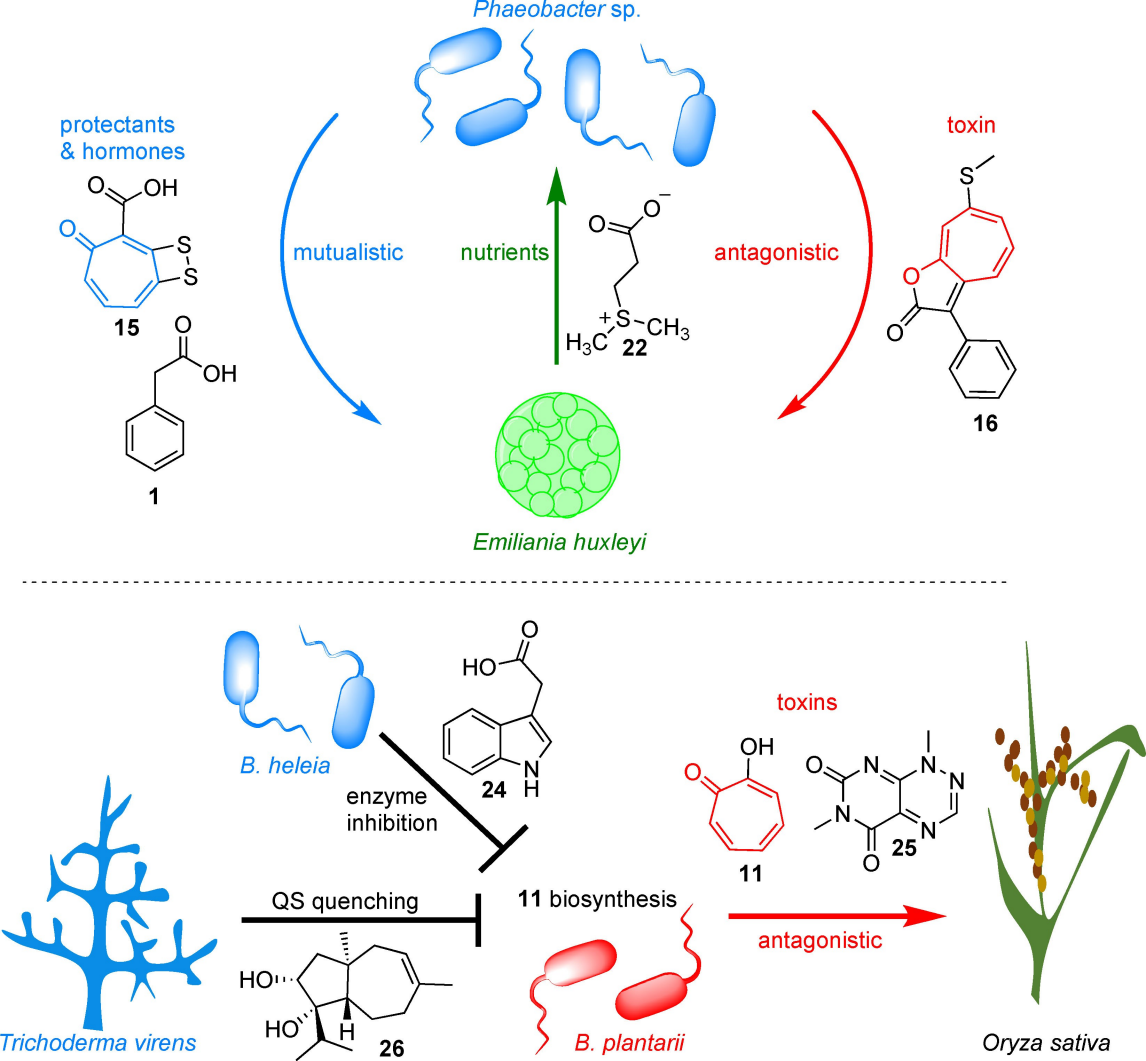
Examples of marine (top) and terrestrial (bottom) symbioses involving bacterial tropone natural products. Top: Dynamic “Dr. Jekyll and Mr. Hyde” symbiosis of marine *Phaeobacter* sp. with the microalga *E. huxleyi*. Compounds **15** and **16** play key roles by acting as protective antibiotic and lethal toxin, respectively (panel adapted from Seyedsayamdost et al.).^53^ Compound **15** might also be an important antibiotic in bacterial symbioses with marine invertebrates (corals, sponges, etc.). Bottom: Antagonistic symbiosis of B. *plantarii* with the rice plant (*Oryza sativa*). *B. heleia* and *T. virens* have probiotic activity by suppressing the formation of toxin **11** by *B. plantarii*. See the text for details.

The closely related *Burkholderia glumae*, *B. plantarii* and *B. gladioli* are causal agents of bacterial panicle blight in an antagonistic symbiosis with rice and represent a major threat to global rice production.^36^, ^37^, ^84^ Symptoms of the disease include panicle blight, seedling blight and sheath rot.^36^ It has been show that these pathogenic *Burkholderia* sp. produce **11** as a key virulence factor that is toxic toward rice seedlings^83^ and derived from **1**.^37^, ^85^, ^86^ Notably, **1** serves as growth hormone in plants but can also be generated *de novo* by *B. plantarii* via the shikimate pathway and phenylalanine before further conversion to **11**.^37^ It is assumed that the primary MoA of **11** is due to its metal‐chelating properties (see Section 3.2.) that deprives the plants from essential ferric iron and thereby causes chlorosis, inhibition of root growth, and wilting.^83^, ^85^ **11** could thus potentially fulfill a similar role as siderophores and, for example, also limit the growth of other fungi and bacteria.^83^ Moreover, **11** was shown to act as autoregulatory signaling molecule in *B. plantarii* that promotes the formation of a biofilm. The pivotal role of **11** for pathogenesis is supported by the observation that exogenously administered **11** phenocopied an infection with *B. plantarii*.^83^, ^84^ In addition to **11**, toxoflavin (**25**) serves as phytotoxic virulence factor, possibly by triggering spontaneous NADH‐dependent generation of reactive oxygen species such as superoxide and hydrogen peroxide (Figure 12).^87^

Interestingly, the **11**‐tolerant *B. heleia* PAK1‐2 strain (isolated from the rice rhizosphere) was found to be an effective biocontrol agent and suppressed symptoms of rice seedling blight disease caused by *B. plantarii*. It was furthermore demonstrated that this effect is not due to interference with *B. plantarii* growth *per se*, but rather by generating the **1**‐analogue indole‐3‐acetic acid (**24**; a plant growth factor) that inhibited the biosynthesis of **11** (Figure 12).^37^ Presumably, this effect is due to the inhibition of one or several of the early **1**‐catabolic enzymes required for **11** biosynthesis. Other analogues of **1** had similar antagonistic effects on the biosynthesis of **11** by *B. plantarii*, for example, (*p*‐isopropylphenyl)acetic acid.^37^ In addition to *B. heleia* PAK1‐2, the **11**‐tolerant ascomycete *Trichoderma virens* PS1‐7 was shown to thwart the biosynthesis of **11** by *B. plantarii* and thus act as another biocontrol agent.^86^ Notably, the underlying inhibition mechanism is decisively different and was shown to depend on the sesquiterpene carot‐4‐en‐9,10‐diol (**26**) in a dose‐dependent manner. Compound **26** normally functions as a critical signaling molecule in *T. virens* PS1‐7 that is produced in the presence of catechol.^86^ In *B. plantarii*, **26** exerts its function via transcriptional suppression of an AHL synthase, which accordingly results in the quenching of quorum sensing (QS) signaling and thus repression of **11** biosynthesis (Figure 12). Surprisingly, a biofilm was still formed (induced by **26**), however, with a defective matrix that lacked fibrous elements and caused reduced cell viability (44 % when induced by **26**‐as compared to 78 % when induced by **11**).^86^ These biofilm defects could be restored by addition of exogenous **11**.^86^ The exact underlying mechanisms, however, remain to be elucidated. These examples indicate that tropones may play a larger role in complex and intertwined symbiotic relationships between plants, antagonistic and mutualistic symbionts and highlight the potential of biocontrol agents for combatting diseases such as bacterial panicle blight.

### 4.2 The role of tropodithietic acid and members of the *Rhodobacteracea* in marine symbioses

#### 4.2.1 Tropodithietic acid producers, host organisms, and symbiotic relationships

The metabolically versatile **15**‐producing *Roseobacter*, *Phaeobacter*, and *Pseudovibrio* are highly abundant and ubiquitous in the marine environment.^11^, ^88^, ^89^, ^90^ For example, the *Roseobacter* lineage encompasses up to 25 % of marine microbes and plays a predominant role in the global carbon and sulfur cycles.^91^ They are present in all major niches, including, for example, seawater, sediments, or microbial mats.^91^, ^92^ Often, these bacteria live associated with marine invertebrates such as ascidians/tunicates,^93^, ^94^ micro^51^, ^53^ and macro algae,^68^ soft and stony corals,^73^, ^95^ tube worms,^96^ numerous marine sponges,^97^, ^98^, ^99^, ^100^, ^101^, ^102^, ^103^ and many other marine eukaryotes.^104^ In particular, the tendency to form strong biofilms also on biotic surfaces is a crucial prerequisite for host colonization.^4^, ^105^ While the type of symbiosis (antagonistic, commensal, or mutualistic) is often challenging to determine and may even be situative (see Section 4.3.), most studies suggest beneficial effects of **15**‐producers on the hosts, presumably by serving as defensive tool against (opportunistic) pathogens such as *Vibrio* spp. Recent studies remarkably showed that the cytotoxic effects of **15** against eukaryotic cells are dampened by **22** (produced, e. g., by corals and algae), suggesting that it may not only serve as food for bacteria but also to protect the hosts themselves from their **15**‐synthesizing microbial symbionts (see also Section 5.1.2.).^106^ In the following sections, we will highlight studies that imply a potential role of **15** in mutualistic symbiotic relationships.

#### 4.2.2 Corals

Coral‐associated bacterial communities have been intensively studied,^107^ yet their ecological role only begins to emerge. For example, cyanobacteria are likely involved in nitrogen fixation to support host growth,^108^ whereas other bacteria (e. g., *Roseobacter* spp. and *Pseudovibrio* spp.) use **22**, which is abundantly produced by corals and might be a key metabolite for the structuring of coral‐associated bacterial communities.^58^, ^109^ It is assumed that symbiotic bacteria also contribute to the host's defenses against coral pathogens, for example, by occupying surface space of the corals or via production of bioactive natural products, yet direct evidence for this is lacking.^73^, ^110^

*Pseudovibrio* spp. appear to be commonly associated with corals and are well known for their adaptation to a symbiotic lifestyle with marine invertebrates.^88^, ^103^, ^111^ This includes various mechanisms for interaction with their hosts, such as evasion of host defense and digestion as well as features allowing for both intra‐ and extracellular colonization.^88^, ^104^, ^112^, ^113^ A recent survey suggested up to 44 % relative abundance in natural (i. e., non‐enriched) bacterial communities, for example, in the stony coral *Stylophora pistillata* and many others.^104^ Additionally, pyrosequencing also verified *Pseudovibrio* spp. in different coral species.^114^ Recently, it was shown that *Pseudovibrio* sp. P12 (isolated from the reef‐building coral *Pocillopora damicornis*), produced **15** with potent activity against invasive coral pathogens such as *V. coralliilyticus* that is resistant to many other antibiotics and *V. owensii* (MICs of 2.3 μM, see also Section 3.1.2.),^73^ which cause progressive tissue loss leading to white bands of exposed coral skeleton (white syndrome). However, it could not be verified that **15** is indeed produced in corals after analyzing individual specimen of four coral species (*P. damicornis*, *Montipora aequituberculata*, *M. turtlensis*, and *Porites cylindrical*).^73^ This might not necessarily mean that **15** is dispensable for coral survival but, for example, could also be due to low abundance in coral tissue or sampling/detection issues.^73^ Another plausible scenario is that **15**‐production is regulated in symbiotic *Pseudovibrio* spp. and depends on chemical cues from invading pathogens.

Interestingly, the inhibitory activity of *Pseudovibrio*. sp. P12 against *V. coralliilyticus* is strongly diminished at higher temperatures, which was concluded not to result from thermal inactivation of **15** but most likely from decreased production levels.^73^ However, contradicting experiments with supernatants of *P. piscinae* 27‐4 cultures pointed to a thermal inactivation and thus temperature‐dependent bioactivity of **15**.^105^ In any case, the decrease in **15** production at elevated temperatures may conceivably be a contributing factor to the onset of white syndrome that is clearly favored under coral heat stress.^73^, ^115^ Summed up, **15** represents a rare case of an antibiotic produced by coral‐associated bacteria with confirmed activity against coral‐associated pathogens.^73^ Further studies are required to clarify if *Pseudovibrio* sp. P12 and related strains are more widespread in corals and whether **15** is indeed produced in the corals as defensive tool against pathogens as part of a symbiotic relationship.

#### 4.2.3 Sponges

Marine sponges are well known to host a substantial amount of phylogenetically diverse microbes that may comprise up to 35 % of their biomasses.^116^ Members of the genus *Pseudovibrio* also appear to play vital roles^88^ and antibacterial activity in sponges has regularly been linked to the presence of *Pseudovibrio* spp..^97^, ^98^, ^99^, ^100^, ^102^ However, it is difficult to assess the true relevance and occurrence of bacteria by culture‐dependent techniques, as numerous strains will not grow under laboratory conditions, which may significantly bias the results.

A steadily increasing amount of evidence, however, suggests that *Pseudovibrio* spp. represent true mutualistic symbionts of sponges. For instance, culture‐independent approaches (e. g., pyrosequencing and fluorescence in situ hybridization (FISH)) verified the presence of *Pseudovibrio* spp. in sponges such as *Aplysilla rosea,* in which *Pseudovibrio* represented the predominant genus and comprised approximately 8 % of the bacterial community.^104^, ^113^, ^117^, ^118^ A recent analysis of 16S rRNA amplicon data from natural bacterial communities of marine invertebrates also corroborated that *Pseudovibrio* spp. occur sponge‐associated, often with a relative abundance of ≥0.1 % of the bacterial communities and in some cases of ≥0.5 %. Not surprisingly, *Pseudovibrio* spp. were also found in some tunicates and, as stated above, with surprising abundance in numerous corals.^104^ Interestingly, *Pseudovibrio* were detected in the sponge *Rhopaloeides odorabile* but not in the surrounding sea water,^113^ while *Pseudovibrio*‐like bacteria were also observed in the larvae of *Mycale laxissima*.^118^ This points to an intimate and specific association with the host and possible vertical transmission of the bacteria, although horizontal acquisition via filtration of sea water cannot be ruled out.^118^ It was also shown that *Pseudovibrio* spp. were absent in diseased sponge specimens but present in healthy ones.^68^, ^119^ For instance, the loss of a strain that is closely related to *Pseudovibrio denitrificans* (referred to as “Alphaproteobacterium strain NW001” in Webster et al.^119^ and capable of producing **15**)^68^ concurred with the ultimately lethal colonization of the sponge by putative pathogens.^68^, ^119^

Aside from **15**, a few additional bioactive natural products have been reported from *Pseudovibrio* spp., that is, heptylprodigiocin,^94^ pseudovibrocin,^73^ 2‐methylthio‐1,4‐naphthoquinone,^120^ and derivatives of di(1*H*‐indol‐3‐yl)methane^121^ as well as tetra(indol‐3‐yl)ethanone.^122^ In several cases, however, **15**‐production has been clearly shown to account for the antibacterial activity of *Pseudovibrio* spp. under laboratory conditions. For example, 26 out of 33 isolated groups of *Pseudovibrio* spp. (obtained from the marine sponges *Axinella dissimilis*, *Polymastia boletiformis* and *Haliclona simulans*),^123^ inhibited *S. aureus* NCDO 949. Moreover, this inhibitory activity could be linked to the formation of **15** in these strains. Interestingly, 93 % (126 out of 136) of all investigated putative sponge symbionts from the deep sea sponges *Hexactinellida* spp. and *Poecillastra* spp. comprising phylogenetically diverse bacterial isolates proved resistant against **15** when spot‐plated and overlaid with the most potent inhibitory *Pseudovibrio* strain from this study.^123^ This possibly implies that natural sponge symbionts may be regularly exposed to **15** and thus acquired resistance mechanisms and furthermore lends support to the hypothesis that **15** protects the sponge from pathogens. Further studies are required to evaluate the degree of resistance and whether it relies on a similar mechanism than described for the natural producers (see Section 3.1.3.).

Summed up, *Pseudovibrio* spp. might be important symbionts of marine sponges and possibly shape the host's microbiota by suppressing the growth of potential pathogens and competitors. However, none of these studies so far addressed whether **15** is truly produced in sponges and its role under natural conditions remains to be conclusively shown. A strong indication that **15** is a relevant antibiotic in naturally‐occurring symbioses may come from studies with oysters and aquacultures that showed a more direct link between the production of **15** and suppression of pathogens (see Sections 4.2.4. and 4.2.5.).

#### 4.2.4 Algae and dinoflagellates

Compound **15** was identified to be responsible for the antibacterial activity of the marine epiphytic bacterium *Pseudovibrio* sp. D323 (and closely related sponge‐associated strains), which was isolated from the red alga *Delisea pulchra*.^68^, ^124^ Once more, the data imply that the potent broad spectrum antibiotic **15** may protect the algae from unwanted surface colonizers and potential pathogens. Accordingly, **15** efficiently suppressed the growth of the pathogenic α‐proteobacterium *Nautella* sp. R11, which forms biofilms on *D. pulchra* and causes bleaching disease.^125^ Hence, **15** production may assist in the chemical defense against this pathogen that furthermore relies on QS‐interfering furanones produced by the algae themselves.^125^ Interestingly, at elevated temperatures, furanone production is reduced,^125^ as well as the efficacy of **15**‐producing bacteria (see Section 4.2.1.). It is therefore plausible that both effects exacerbate the disease and lead to a higher susceptibility for infection.

In addition to macroalgae, **15**‐producing bacteria, such as *Ruegeria sp*. TM1040 (formerly *Silicibacter*), are often associated with **22**‐producing microalgae (i. e., haptophytes/prymnesiophytes, see Section 4.3.) and dinoflagellates such as *Pfiesteria* spp., *Prorocentrum* spp., and *Alexandrium* spp.^4^, ^126^ The symbiosis with dinoflagellates involves bacterial chemotaxis toward **22** and eventually results in the settling of the bacteria and biofilm formation on the host. Compound **22** can then be catabolized by the bacteria via 3‐methylmercaptopropionate (Figure 10).^127^ Biofilm formation and **15**‐production were correlated in *Ruegeria sp*. TM1040, thus implying a role for **15** in the symbiotic relationship.^4^ Accordingly, **15** may impede the growth of other microbes and potential pathogens on the dinoflagellates and thus promote the growth and survivability of *Ruegeria sp*. TM1040.^4^

#### 4.2.5 Mollusks

Bacterial pathogens not only pose a severe threat in the natural environment for shellfish such as oysters or scallops, but also in aquacultures (see Section 5.2). Recently, P. *inhibens* S4 was isolated from the surface of the inner shell of a healthy oyster.^128^ Similarly, a P. *inhibens* strain was obtained from both aquacultured and collected scallops (*Pecten maximus*),^129^ thus suggesting natural symbiotic relationships between these mollusks and the bacteria. Recent studies verified that the oyster‐associated P. *inhibens* S4 protects the larvae and juveniles of Pacific oysters (*Crassostrea gigas*) and significantly reduces mortality rates when exposed to several pathogenic bacteria.^130^ Zhao et al.^74^ showed that pre‐colonization by *P. inhibens* S4 and the ability to form biofilms are important to protect oyster larvae and that the primary probiotic inhibitory effects can most likely be attributed to the presence of **15**. Biofilm formation may allow *P. inhibens* S4 to grow on oyster larvae and thus impede colonization by pathogens while also triggering QS‐mediated biosynthesis of **15** (see also Section 4.4.).^74^ Taken together, the production of **15** by natural probiotic bacterial colonizers of oysters such as *P. inhibens* S4 seems to be pivotal for host health.

### 4.3 Chemical control of a dynamic symbiosis between microalgae and bacteria by tropodithietic acid and roseobacticides

Both **15** and roseobacticides are crucial for a symbiotic relationship between *Phaeobacter* sp. and its host, the pelagic marine microalgae *E. huxleyi* that is a major contributor to global O_2_ production by photosynthesis.^131^ In addition, photosynthesis sequesters CO_2_, while the formation of coccolithophores, (i. e., CaCO_3_ disks) releases CO_2_ by decreasing the alkalinity and increasing the *p*CO_2_ of surface water and the atmosphere (referred to as the “biological carbonate pump”: Ca^2+^+2 HCO_3_^−^→CaCO_3_+CO_2_+H_2_O).^132^ The haptophyte *E. huxleyi* thus plays a major role in the global carbon cycle and is an important primary producer of organic molecules in the ocean. This role is emphasized by the fact that *E. huxleyi* is widespread and can form enormous blooms (e. g., 250.000 km^2^ with high cell densities),^133^ which are even detectable by satellites.^134^ *E. huxleyi* can make up to 80–90 % of eukaryotic cells in phytoplankton blooms, whereas members of the *Rhodobacteracea* are predominant among bacteria (up to 60 % of the bacterial cells).^53^, ^135^

*E. huxleyi* was furthermore shown to associate with *Phaeobacter* sp. in a remarkable dynamic symbiosis.^53^ In the mutualistic phase the host *E. huxleyi* provides carbon and energy sources as well as a surface for colonization, while the bacteria in turn generate growth hormones (in the form of **1**) and antibiotics (in the form of **15**) for defense against potential pathogens. However, when the algae senesce, an antagonistic phase is prompted, in which *Phaeobacter* sp. generate algaecidal roseobacticides (such as **16**) that kill the host (Figure 12).^53^ It could furthermore be shown that *p*‐coumaric acid produced by the algae induces roseobacticide formation and is key for switching between symbiotic phases. *p*‐coumaric acid is an unusual breakdown product of the *E. huxleyi* cell wall and represents a chemical cue of dying host cells that triggers roseobacticide biosynthesis.^51^, ^52^, ^53^ This results in a rapid collapse of the bloom and thus provides abundant access to food sources, which support bacterial growth, dissociation from the host and ultimately dispersion of *Phaeobacter* sp. (see also 4.4). Remarkably, *p*‐coumaric acid was furthermore demonstrated to not only act as signaling molecule, but also as precursor for Tyr formation that is incorporated into roseobacticide A.^52^ In addition to *p*‐coumaric acid, ferulic acid, cinnamic acid, and particularly sinapic acid triggered formation of roseobacticides. This rather spectacular symbiotic interplay mediated by two biosynthetically related bacterial natural products with distinct bioactivities was aptly referred to as the “Dr. Jekyll and Mr. Hyde chemistry” of *Phaeobacter* sp.^53^

### 4.4 Tropodithietic acid as quorum sensing signal

Compound **15** appears to adapt important roles as virulence factor and antibiotic in terrestrial and marine environments and surprisingly also as QS signal, as shown by transcriptome analysis and phenotypic screenings of *P. inhibens*.^136^ QS is a means of communication between bacterial cells mediated by autoinducers such as *N*‐acyl−homoserine lactone (AHL) that control gene expression. AHL binds to LuxR‐type transcriptional regulators (“AHL regulators”), which in turn control gene expression often on a global scale. QS thus enables a group‐like behavior that is directly affected by the cell density and may involve, for example, biofilm formation, cell motility, or secondary metabolism. Remarkably, it could be shown that exogenous **15** affected expression of ≈10 % of the *P. inhibens* genes in a LuxR‐dependent manner similar to AHL. Moreover, **15** triggered a QS response at concentrations of 1.5 μM, which is lower than most MICs of **15** against bacteria that typically range between 1–120 μM (however, not >100‐fold lower in general as stated in Beyersmann et al.,^136^ as the alleged typical MICs of 188.5 μM to 5.9 mM for **15** that were cited from Porsby et al. were in fact determined with impure extracts and are thus much higher than MICs of purified **15**, see also Section 3.1.2.).^67^

**Figure 13:**
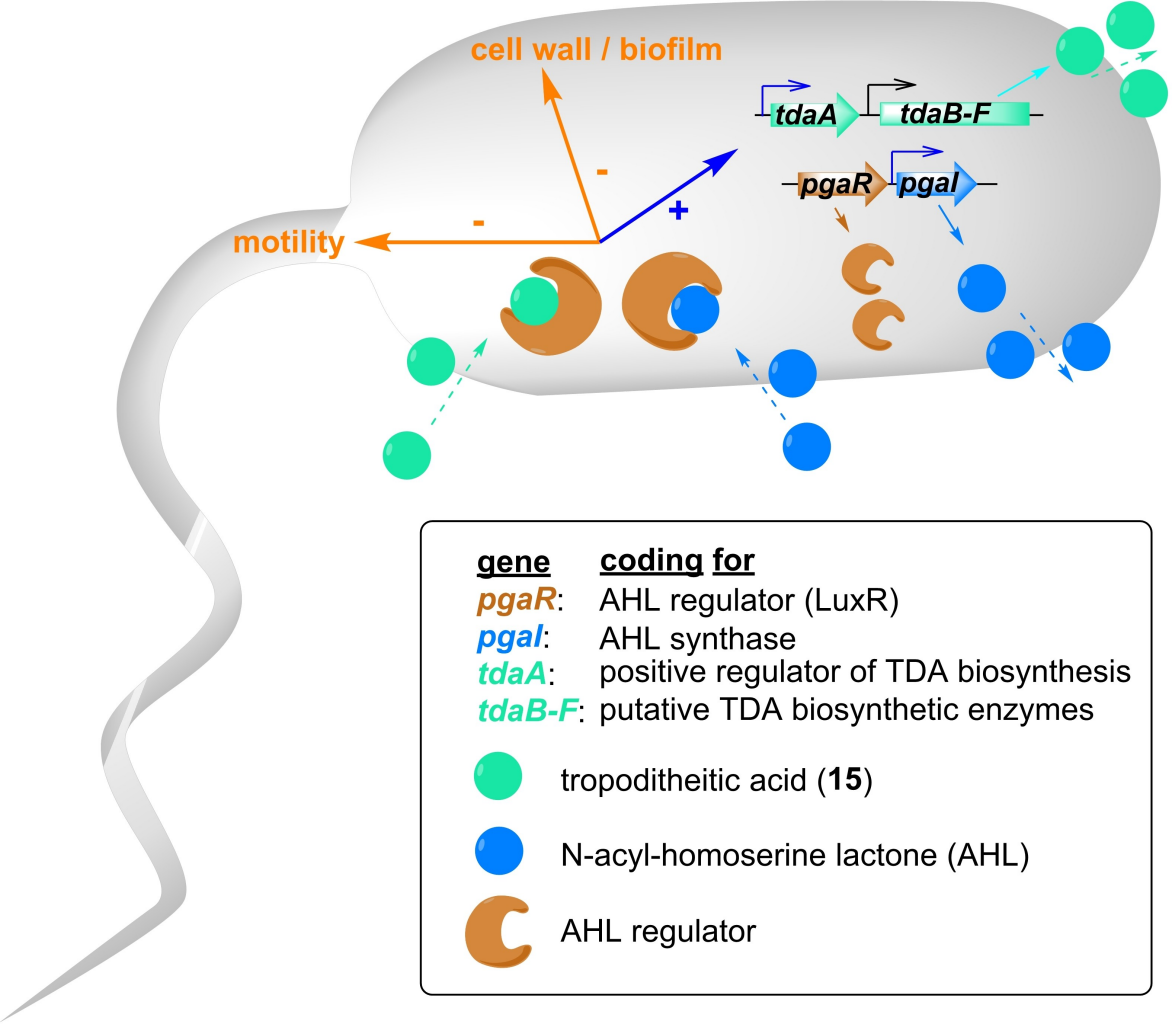
Proposed role of **15** as quorum‐sensing signal in *P. inhibens* (modified after Beyersmann et al.),^136^ resulting in a double positive feedback loop (see text for details). Note that the exact interaction of **15** with the AHL regulator remains to be elucidated.

Interestingly, **15** not only induced expression of the genes encoding the LuxR‐type AHL regulator and the AHL synthase but also of the *tda* cluster including *tdaA* (that encodes a positive regulator of **15** biosynthesis) and thus boosts its own production in a double positive feedback loop (Figure 13). Hence, **15** may represent a rare case of a natural product that simultaneously serves both as antibiotic and as global gene regulator,^136^ which may also reduce the energetic costs for its biosynthesis.^136^ Note that the exact interaction of **15** with the LuxR‐type transcriptional regulator requires further investigation. It is noteworthy that a recent study with sponge‐isolated *Pseudovibrio* sp. showed neither significant (auto)induction of **15** biosynthesis by 1 μM of **15** nor by 100 nM of the QS signal *N*‐3‐hydroxydecanoyl‐l‐homoserine lactone.^123^ As can be expected, this implies that the underlying regulation mechanisms for **15** biosynthesis are not universal in bacteria.

The **15**‐triggered changes in gene expression in *P. inhibens* eventually led to a decrease in surface attachment (i. e., proclivity to form biofilms) and in cell motility (flagella formation). It was proposed that the QS role of **15** may maintain the ability of *P. inhibens* and other producers to disperse and attach to new abiotic surfaces and host organisms, for example, in the form of “hitchhiking” on phytoplankton for rapid spreading of the bacteria.^136^ Notably, it appears counterintuitive at first that flagella formation is downregulated upon switching from sessile to pelagic lifestyle. However, flagella were previously shown to play essential structural roles in biofilms by wrapping around cells to bind them tightly together^137^, ^138^ and are most likely important during all stages of biofilm formation.^138^ In addition, flagella adopt a pivotal mechano‐sensory role during initial surface adhesion for biofilm formation.^139^ Hence, we speculate that flagella in *P. inhibens* and other marine bacteria that switch from pelagic to sessile lifestyles may also be critical for surface attachment and biofilm stability. On the other hand, by decreasing the abundance of flagella, the biofilm is weakened as prerequisite for efficient dispersion.

Finally, during the switch from mutualistic to antagonistic behavior of *Phaeobacter* sp. in the symbiosis with *E. huxleyi* 53 (see Section 4.3.), bacterial growth may be drastically increased due to nutrient release upon algae death, presumably triggering QS and eventually renewed **15** production to promote dispersion and ultimately settlement on new hosts.^136^ In addition, **15** may not only serve as protection against pathogenic bacteria during the mutualistic phase but even be part of a chemical trap. As marine bacteria are often attracted to algal **22**,^140^ Wilson et al.^63^ speculated that the concurrent formation of chemoattractant **22** and antibiotic **15** could lure other microorganisms to the symbiotic consortium between algae and the **15**‐producing bacteria. After death of the invading microorganisms caused by **15**, important scarce nutrients such as phosphorus or iron could be sequestered.

## 5 Biotechnological and Medical Applications

### 5.1 Tropodithietic acid as potential antibiotic or anti‐cancer drug

Natural products from microbial symbionts of eukaryotic hosts are presumably less toxic against other eukaryotes and are thus considered promising candidates for drug development. A few recent studies summarized in the following sections show good promise for the usage of **15** as antibiotic or anti‐cancer drug that has high potency against numerous bacteria as well as cancer cell lines. Additional extensive studies would be required to further assess the potential of **15** or related compounds.

#### 5.1.1 Development of resistance

Antibiotic resistance of pathogenic bacteria is a major and rapidly increasing threat to public health and safety. Hence, the apparent negligible and slowly developing tolerance of bacteria against **15** is a promising feature.^67^ Accordingly, single exposure to **15** (using different methods of administration) did not result in significant resistance in all tested bacterial strains. This included the medically problematic *Salmonella enterica* serovar Typhimurium SL1344 that harbors the multidrug efflux pump AcrB. Similarly, mutant screening of a transposon library did not yield a resistant strain. Moreover, attempts to gradually adapt bacteria to steadily increasing levels of **15** (in >300 generations), only resulted in low levels of tolerance (2x more than the MIC), which, however, were rapidly lost after only one passage in **15**‐free medium.^67^ Hence, the tolerance was likely not caused by a true adaptation on a genetic level.^67^ This is not surprising with respect to the MoA of **15** that involves the disruption of the PMF (see Section 3.1.1), because a resistance against such a highly conserved and indispensable process is unlikely to develop through basic mutations.^63^ Rather than being overwhelmed by sudden exposure to a lethal concentration, the incremental increase of **15** in the cultures may have allowed the bacteria to induce and maintain a proper acid/oxidative stress response. Interestingly, *S*. Typhimurium mutants in which genes coding for efflux pumps and porins were inactivated, did not show increased susceptibility to **15** compared to the wild type, implying that these components do not play a role in resistance.

#### 5.1.2 Toxicity of tropodithietic acid and protective effects of DMSP

Another auspicious feature of **15** is the potency against cancer cell lines, in contrast to less susceptible noncancerous epithelial cells (see Section 3.1.2).^63^ However, recent studies showed significant toxicity against neuronal cells (N2a and OLN‐93) that were highly susceptible to **15**, that is, a concentration of 1.4 μM induced morphological changes, while 2.4 μM of **15** caused severe cytotoxicity. This involved detrimental effects on mitochondria and cytoskeletal elements (microtubules and microfilaments) and led to increased Ca^2+^‐influx. Moreover, stress activated MAP kinases ERK1/2 and the heat shock protein Hsp32/HO‐1 (i. e. induced under oxidative stress) were upregulated. The stress responses, however, were not sufficient to protect the cells from death.^141^ The neuronal cells were apparently even more susceptible to **15** than different carcinoma cell lines (showing cytotoxicity at concentrations of >38 μM).^141^

A later study by the same group remarkably showed that **22** is likely anti‐oxidative and protects cells to some extent against the toxic effects of **15**.^106^ As dimethyl sulfone (that is structurally similar to **22**) can cross the blood‐brain barrier and because up to 5 mg/mL of **22** showed no cytotoxicity against eukaryotic cells but rather acted neuroprotective in rodents,^142^ it may be worthwhile to further study the effects of **22** in combination with **15**. It would be interesting to see if, for example, if **15** still maintains anti‐cancer and/or antimicrobial activity in presence of **22** (also from an ecological perspective regarding symbiotic relationships of **15**‐producing bacteria with **22**‐generating marine invertebrates and algae, see 4.2). Notably, despite these adverse effects on neuronal cells, a negative impact has yet to be reported by **15**‐producing bacteria in aquacultures for fish and mollusks (see 5.2). Moreover, in contrast to other probiotic bacterial candidates that were tested, no detrimental effects were observed upon exposure to **15**‐producing *P. inhibens* DSM17395 and *R. mobilis* F1926 in the eukaryotic worm *Caenorhabditis elegans* (a model system for testing cytotoxicity) as well as in brine shrimps (*Artemia* sp.).^143^ Particularly noteworthy is the fact that fish larvae are protected from pathogens by probiotic **15**‐producers without noticeable negative effects on their development and health (see Section 5.2),^130^ which suggests that their neuronal cells remain unaffected. As a logical next step, **15** should be tested in an *in vivo* mammal model to better assess the efficacy and hazards of a topic or systemic treatment against cancer or infection.

### 5.2 Probiotic *Rhodobacteraceae* in (shell)fish and coral aquacultures

The aquaculture industry for fish or shellfish is rapidly growing and becomes increasingly important,^144^ as it presents an alternative to conventional fishing practices and might prevent detrimental overfishing. Bacterial pathogens, however, pose a major threat and often cause severe disease and mortality, which in turn results in vast economic losses. Rotifers are an important food source for fish in aquacultures, while microalgae in turn serve as food for the rotifers and thus promote the growth and survivability of the fish larvae. This results in an enrichment of organic nutrients that further favors opportunistic pathogens such as *Vibrio* spp..^130^ Particularly susceptible to infection are larvae of fish and invertebrates that cannot be vaccinated^144^ and are threatened, for example, by *V. tubiashii* RE22, *Roseovarius crassostreae* CV919, and *V. anguillarum* NB10. *V. tubiashii* infects larvae of bivalve mollusks and is responsible for invasive and toxigenic disease, which causes drastic mortalities in *C. gigas* aquacultures. *R. crassostreae* causes juvenile oyster disease that results in high mortalities among eastern oysters (*Crassostrea virginica*) at water temperatures ≥20 °C with significant economic and ecological relevance, while *V. anguillarum* is a typical fish pathogen that causes vibriosis, for instance in cod or turbot larvae.^130^

The past and ongoing excessive (mis)use of persistent antibiotics in aquacultures not only negatively affects the natural microbiota, but also caused a surge in resistant strains and thus poses a severe threat to the environment and to human health.^145^ Hence, alternative remedies such as probiotic bacteria are required to counteract the onset of bacterial diseases and decrease mortality rates.^74^ In that regard, **15**‐producing members of the *Rhodobacteraceae* (e. g., *Phaeobacter* spp. and *Ruegeria* spp.) are often found in marine rearing sites of larvae or fish and appear highly promising.^90^, ^144^ Among the salient features of **15** are the high potency against common marine bacterial pathogens such as *Vibrio* spp. (Section 3.1.2.), negligible development of resistance (Section 3.1.3.), good degradability, and lacking toxicity toward (in)vertebrates and their larvae when administered as probiotics (Section 5.1.2.). For example, *P. inhibens* efficiently suppressed V. anguillarum and prevented vibriosis in cod larvae, thus drastically increasing survival rates.^130^ *P. inhibens* DSM 17395 successfully eradicated the rifampicin resistant *V. vulnificus* CMCP6 in an co‐culture oyster model system in contrast to a **15**‐deficient *P. inhibens* mutant.^72^ In addition, **15**‐producing *Phaeobacter* spp. and *Ruegeria* spp. (unlike nonproducers) inactivated both pelagic and sessile *V*. anguillarum in model systems for fish larval aquaculture^144^ and proved antagonistic in a rearing farm for danish turbots (Scophthalmus maximus) by colonizing distinct niches (*Phaeobacter* spp. were dominant in fish/larvae/zooplankton tanks while *Ruegeria* spp. were found in the algal cultures).^90^ Moreover, *P. inhibens*, successfully antagonized *V. anguillarum* in cultures of microalgae (*Tetraselmis suecica* and *Nannochloropsis oculata*) and rotifers (*Brachionus plicatilis*). These ostensibly simple and unproblematic co‐cultivations with both the microalgae and the rotifers thus represent additional advantages when applying **15**‐producing bacteria in aquacultures. The same study also showed that P. *inhibens* prevented vibriosis in cod larvae, whereas, once more, a **15**‐deficient mutant strain failed.^130^

Similarly, *P. inhibens* S4 protected larval and juvenile *C. virginica* from pathogenic *R. crassostreae* and *V. tubiashii* depending on the production and secretion of **15**,^74^ which did not negatively affect oyster survival.^74^, ^128^ After removing the probiotics, however, this protection was only maintained for additional 24 h.^128^ This probiotic effect was investigated using competition assays against *V. anguillarum* NB10, *V. tubiashii* RE22 and *Roseovarius crassostreae* CV919, as well as a test system for oyster larvae survival in presence of *V. tubiashii*. The effects of both biofilm formation and **15** production on the probiotic activity of *P. inhibens* S4 were separately investigated, showing that **15** formation is indispensable for the probiotic effect and more important than biofilm formation. To achieve that, specific genes were inactivated that were either required for **15** biosynthesis (resulting in completely abolished **15** production) or biofilm formation (decreasing biofilm formation by ∼60 % compared to the wild type level). While growth rate or final cell density in bacterial liquid cultures were not affected in these mutant strains,^74^ both were differently impaired in their ability to protect oyster larvae from *V. tubiashii*. The **15**‐deficient mutant neither inhibited planktonic *V. tubiashii* nor prevented biofilm formation on a glass coverslip. Also, no inhibition zones were detected on agar plates when *V. anguillarum* NB10, *V. tubiashii* RE22, and *R. crassostreae* CV919 were exposed to this mutant. Interestingly, supplementation of the culture with **15** inhibited planktonic *V. tubiashii* but did not affect already aggregated cells in a biofilm. In contrast, the biofilm‐impaired mutant that showed normal levels of **15** production merely exhibited reduced and delayed inhibitory effects against pathogens in co‐colonization experiments.^74^ Also, the loss of probiotic activity against *V. tubiashii* in the oyster larvae survival assays was less drastic compared to the **15**‐deficient mutant.^74^ A sufficient time period (24 h) proved important to allow pre‐colonization by *P. inhibens* S4, which more severely affected the much faster growing *V. tubiashii* RE22 (both in planktonic and in sessile, biofilm form) as compared to simple co‐culturing.^74^ Future studies should investigate whether the probiosis is based on the antibiotic activity of **15** alone or also caused by its signaling function.^136^

Possibly, **15**‐producing *Rhodobacteraceae* could also prove useful for coral aquaculturing, which becomes increasingly important for the aquarium trade, the pharmaceutical industry, as well as for the replenishment of damaged coral reefs.^146^ To our knowledge, no studies in this direction were conducted so far, yet it appears plausible that cultured corals and their larvae could be protected by probiotic bacteria similar to fish and molluscs, also because **15**‐producing *Pseudovibrio* spp. are naturally associated with these invertebrates and thus unlikely to have a harmful affect (see Section 4.2.1). For the future, it may also be a viable option to optimize **15**‐producing strains via bioengineering, for example, by attempting to realize and combine an increased self‐resistance with overexpression of **15** biosynthetic genes.

### 5.3 Non‐sulfur tropolones

Compound **13** and other (hydroxy‐)tropolones show antimicrobial, antitumor, and antiviral activities, and are potent inhibitors of medically relevant metal‐dependent enzymes (see Section 3.2). Hence, these compounds might also be of interest for further evaluation and development as potential drugs. Compared to *S. tropolofaciens* K611‐97, from which **13** was first isolated,^40^ *S. cyaneofuscatus* Soc7 as well as *S. luteogriseus* Slg41 produced **13** without genetic modification with substantially higher yields (ca. 10,000 times) of 380 and 230 mg/L, respectively. The data suggested that shikimate pathway enzymes (TrlB, TrlH, possibly TrlG) and a transcriptional regulator (TrlR) increased the production of **13** by promoting the *de novo* synthesis of aromatic pathway precursors.^40^ Genetic manipulation of these producers might possibly further improve the yield. Other obvious starting points for bioengineering efforts could be the proposed ring hydroxylases TrlE and TrlCD. For example, the substrate scope or regioselectivity of these enzymes may be targeted, with the aim of generating additional bioactive analogues. Recent studies also aimed at establishing *E. coli* as host for **9** production by combining promotor replacement with inactivation of PaaZ−ALDH to redirect the metabolic flow toward **9** production, achieving titers of up to 65 mg/L, which could serve as starting point for further improvement.^33^

## 6 Summary and Outlook

The biosynthesis of the bacterial tropone backbone is exceptional, as it directly derives from a primary catabolic pathway. Most likely, the spontaneous formation of **8** initially merely represented a metabolic accident. In order to counteract the depletion of the CoA pool by accumulation of **8**, bacteria developed different strategies. This involved, for example, the recruitment of the substrate‐channeling fusion protein PaaZ and of the dedicated **8**‐specific thioesterase PaaY. More intriguingly, some bacteria instead convert **8** into bioactive natural products. In other words, these bacteria “take the good with the bad” and some even devised strategies to boost formation of these natural products, thus turning a catabolic route into a hybrid or even fully anabolic pathway. It is noteworthy that in contrast to typical bacterial secondary metabolic gene clusters that are often found in very few genomes, roughly 16 % of all bacteria possess a **1**‐catabolic gene cluster and thus in principle the required core enzymes for tropone natural product formation.^12^ However, many will use this pathway primarily for aromatic degradation and suppress **8** formation. Genomic analysis may provide important clues, for example, the presence of specific genes required for **15** or **13** formation. Also, genes encoding ring‐cleaving and **6**‐oxidizing enzymes may hint at the proclivity of the respective bacteria for tropone natural product biosynthesis, such as genes encoding additional ECHs or dysfunctional ALDHs.

The seemingly simplistic and minimalistic features of tropone natural products belie their high pharmaceutical potency that rival biosynthetically and structurally more elaborate compounds, such as polyether antibiotics with an analogous MoA to **15**. Moreover, these remarkable small compounds represent key components in mutualistic and antagonistic symbioses that could be pivotal for the survivability of numerous marine invertebrates (e. g., sponges, corals, or molluscs) and algae. Also because of the vast abundance of biofilm‐forming and **15**‐producing bacteria in the oceans, it is distinctly possible that tropone natural products are relevant for shaping microbial communities in various marine habitats. Lastly, the potent antibiotic activity (combined with the apparently slowly developing resistance in the case of **15**) makes tropone natural products promising candidates for biomedical and biotechnological applications, for instance in (shell)fish or coral aquacultures and conceivably even as future antibiotic or anti‐cancer drug.

## Conflict of interest

The authors declare no conflict of interest.

## Biographical Information

*Ying Duan did her Masters from 2013–2016 in the group of Prof. Pengmin Li at the Northwest A&F University in Yangling where she worked on the secondary metabolism of anthocyanin. After that, she started her PhD work under the supervision of Dr. Robin Teufel at the Faculty of Biology of the University of Freiburg. Her project mainly focuses on the investigation of the biosynthesis of tropone bacterial natural products*.

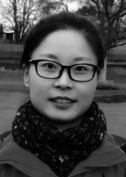

## Biographical Information

*Melanie Petzold née Spieker obtained her diploma in Nanostructure Science from Kassel University in 2014. Directly after, she started to work at the Department of Biology at the St. Francis Xavier University (Antigonish, NS, Canada) under the supervision of Prof. William S. Marshall. Since 2015, she is conducting her PhD studies under the supervision of Dr. Robin Teufel at the Faculty of Biology of the University of Freiburg. Her PhD work will soon be concluded and focusses on the mechanistic and structural investigation of a key enzyme from bacterial phenylacetate catabolism*.

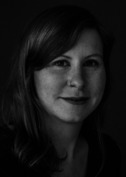

## Biographical Information

*Raspudin Saleem‐Batcha received his Master in Integrated Biology from Madurai Kamaraj University. He then obtained a PhD from University of Lübeck with Rolf Hilgenfeld in 2014 where he worked on crystallographic studies of bacterial stringent factors. In 2015, he joined the Institute of Biology at the University of Freiburg under the supervision of Robin Teufel where he structurally characterized unusual (flavo)enzymes from bacteria. Recently, he joined the group of Jennifer Andexer at University of Freiburg*.

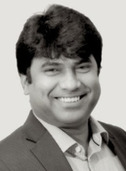

## Biographical Information

*Robin Teufel received his PhD in 2011 in the group of Georg Fuchs (Freiburg) on bacterial aromatic catabolism, before becoming a postdoctoral fellow in the group of Bradley Moore (UC San Diego; 2012–2015), where he investigated complex enzymatic reactions for pharmacophore formation in bacterial polyketide and meroterpenoid biosynthesis. Since 2015, he has been the head of an independent research group in Freiburg funded by the DFG through the Emmy Noether Programme and now the Heisenberg Programme. His work focusses on the enzymology of bacterial primary and secondary metabolic pathways*.